# Metal–Phenolic Network-Facilitated “Foe-to-Friend” Conversion of Melittin for Cancer Immunotherapy with Boosted Abscopal Effect

**DOI:** 10.34133/research.0052

**Published:** 2023-03-08

**Authors:** Yuxin Guo, Xinping Zhang, Shao-Zhe Wang, Hui-Heng Feng, Shun-Yu Wu, Fu-Gen Wu

**Affiliations:** State Key Laboratory of Bioelectronics, School of Biological Science and Medical Engineering, Southeast University, 2 Sipailou Road, Nanjing 210096, P. R. China.

## Abstract

As a naturally occurring cytolytic peptide, melittin (Mel) has strong cytolytic activity and is a potent therapeutic peptide for cancer therapy. However, the serious hemolytic activity of Mel largely impedes its clinical applications. In this work, based on the strong interactions between proteins/peptides and polyphenols, we develop a tannic acid–Fe^3+^ metal–phenolic network (MPN)-based strategy that can convert Mel from foe to friend via shielding its positive charges and reducing its hemolytic activity. Besides, an immune adjuvant resiquimod (R848) is also introduced for immunostimulation, affording the final Mel- and R848-coloaded nanodrug. The Mel-caused membrane disruption can induce immunogenic cell death for immunostimulation, R848 can act as an immune adjuvant to further facilitate the immunostimulatory effect, and the tannic acid–Fe^3+^ MPN-mediated Fenton reaction can produce reactive oxygen species for cancer treatment. Further experiments reveal that the nanodrug can effectively cause immunogenic cell death of tumor cells and arouse robust intratumoral and systemic antitumor immunostimulation. In the bilateral tumor-bearing mouse models, the nanodrug considerably destroys the primary tumor and also boosts the abscopal effect to ablate the distant tumor. Collectively, the MPN-facilitated “foe-to-friend” strategy may promote the practical applications of Mel and foster the development of cancer immunotherapeutics.

## Introduction

Melittin (Mel), a main component of the naturally occurring bee venom, is a cationic and amphiphilic peptide that comprises 26 amino acids (GIGAVLKVLTTGLPALISWIKRKRQQ-NH_2_) [1–3]. Besides, Mel is a nonselective cytolytic peptide, which can destroy all eukaryotic and prokaryotic cytomembranes via forming transmembrane pores, resulting in the increased permeability of cell membrane and cell death [4,5]. Since Mel-induced cancer cell death can disrupt cell membrane and lead to the release of tumor-associated antigens and damage-associated molecular patterns (DAMPs), it is expected that Mel may hold the capacity to cause immunogenic cell death (ICD) of tumor cells for anticancer immunostimulation.

Despite the strong cytolytic activity and superb anticancer performance of Mel, the serious hemolytic activity of Mel largely impedes its clinical applications [6–9]. To suppress the hemolysis of Mel, diverse nanoplatforms have been prepared for Mel delivery [2,4,5,9–16]. Liu et al. [4] used serum albumin-decorated boehmite to load Mel via noncovalent interaction. Yu et al. [5] synthesized phospholipid monolayer to shield the positive charges of Mel and reduce its hemolytic activity. Our group fabricated Mel–photosensitizer nanoassemblies to reduce the hemolytic activity of Mel and further decorated hyaluronic acid on the nanoassemblies to improve their stability [2,9]. Besides, polymeric nanoparticles (NPs) and lipid disks were also fabricated for the delivery of Mel [13–16]. Although these NPs can reduce the hemolysis of Mel, the cytolytic activity and anticancer outcome of the loaded Mel are also largely decreased. Thus, a nanoplatform that can not only mitigate the hemolysis of Mel but also maintain its cancer killing effect is important for the successful clinical applications of Mel.

As a widely used drug carrier, human serum albumin (HSA) has good physiological stability, satisfactory biocompatibility, and tumor-targeting capacity and has been widely used clinically [17–22]. Hence, we wonder if HSA can be applied as a safe and effective nanocarrier for Mel delivery. On the other hand, natural polyphenols, which have universal adherent property, have been widely used to fabricate various NPs [23–28]. In particular, polyphenols can bind with proteins via noncovalent interactions (e.g., hydrophobic interaction, van der Waals force, electrostatic interaction, hydrogen bonding, and π–π stacking) [23,29–32]. Moreover, polyphenols and different kinds of metal ions can form metal–phenolic networks (MPNs), which can coat the surfaces of various NPs to improve their biostability [23,33–42]. Among different kinds of MPNs, the Fe^3+^-containing MPNs can induce Fenton reaction, which converts the intratumoral H_2_O_2_ to highly toxic HO• for chemodynamic therapy [17,25,43–49]. Based on the strong interactions between polyphenols and proteins, we conjecture that polyphenols or MPNs may be applied to stabilize the HSA carrier and interact with Mel to reduce its hemolytic activity.

**Fig. 1.**
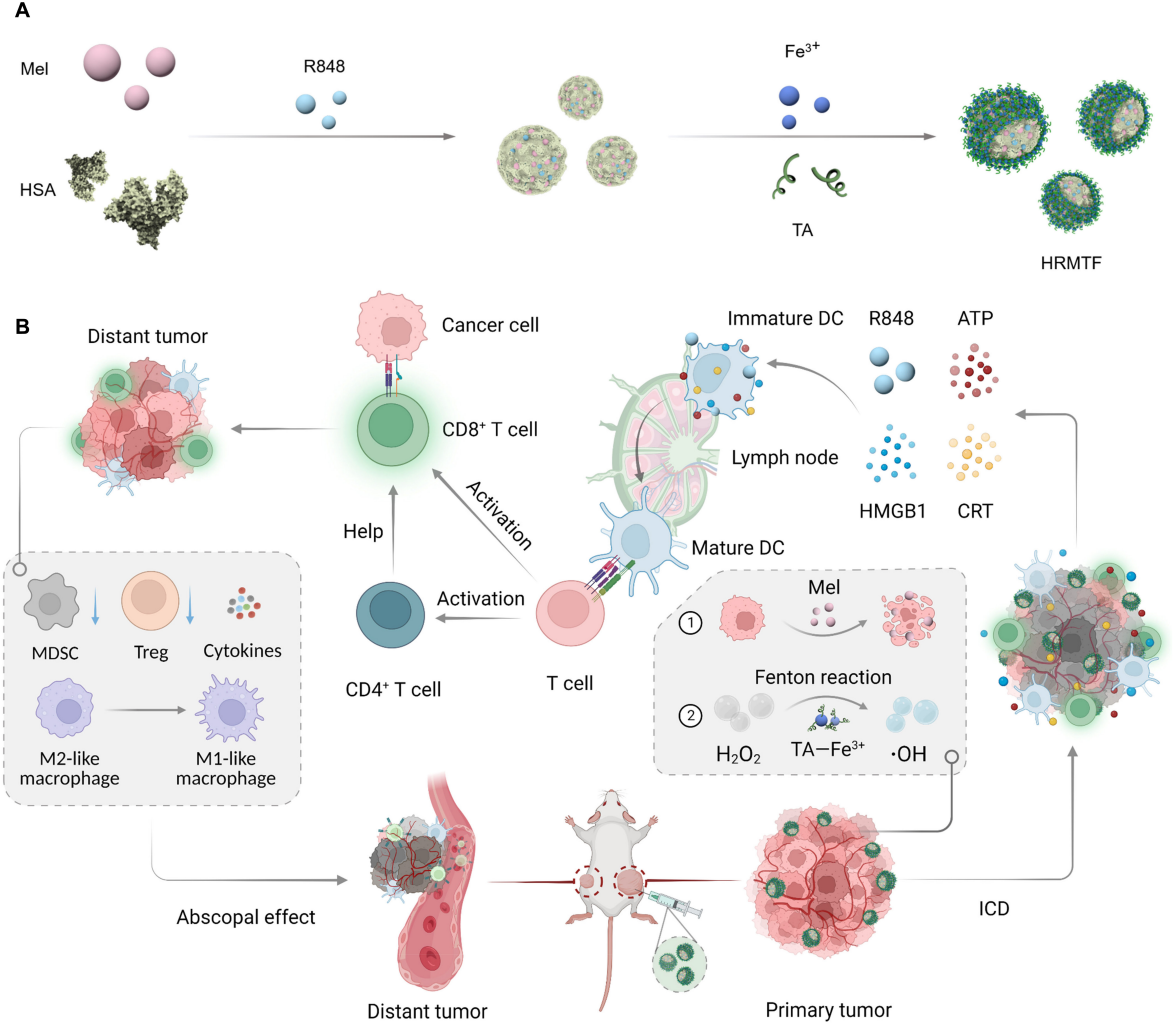
Scheme illustrating the preparation of the HRMTF NPs (A) and the HRMTF NP-induced ICD, immunostimulation, and abscopal effect (B).

In this study, we used HSA and tannic acid (TA, a naturally occurring polyphenol) for Mel delivery (Fig. 1A). HSA, which has good in vivo stability and biocompatibility, was applied as the nanocarrier. TA was introduced to crosslink HSA and Mel and mitigate the hemolytic activity of Mel. Fe^3+^ was further incorporated into the NPs to coordinate with TA to form the MPNs and improve the stability of the NPs (Fig. 1A). An immune adjuvant resiquimod (R848) was also loaded in the NPs for immunostimulation to afford the final product HSA–R848–Mel–TA–Fe^3+^ (termed HRMTF) NPs, which can convert Mel from foe to friend via shielding its positive charges and reducing its hemolytic activity. In the HRMTF nanodrug, the TA–Fe^3+^ MPN can trigger Fenton reaction for cancer chemodynamic therapy. Mel can disrupt cell membrane and induce ICD of cancer cells, and R848 can further stimulate the immune system for cancer immunotherapy. Taken together, the HRMTF NPs can efficiently induce ICD of tumor cells and arouse robust intratumoral and systemic antitumor immunostimulation (Fig. 1B). In the bilateral 4T1 tumor-bearing BALB/c mouse model, the HRMTF NPs can considerably damage the treated primary tumor and also boost the abscopal effect to suppress the growth of the distant tumor (Fig. 1B).

## Results

### Fabrication and characterization of HRMTF NPs

**Fig. 2.**
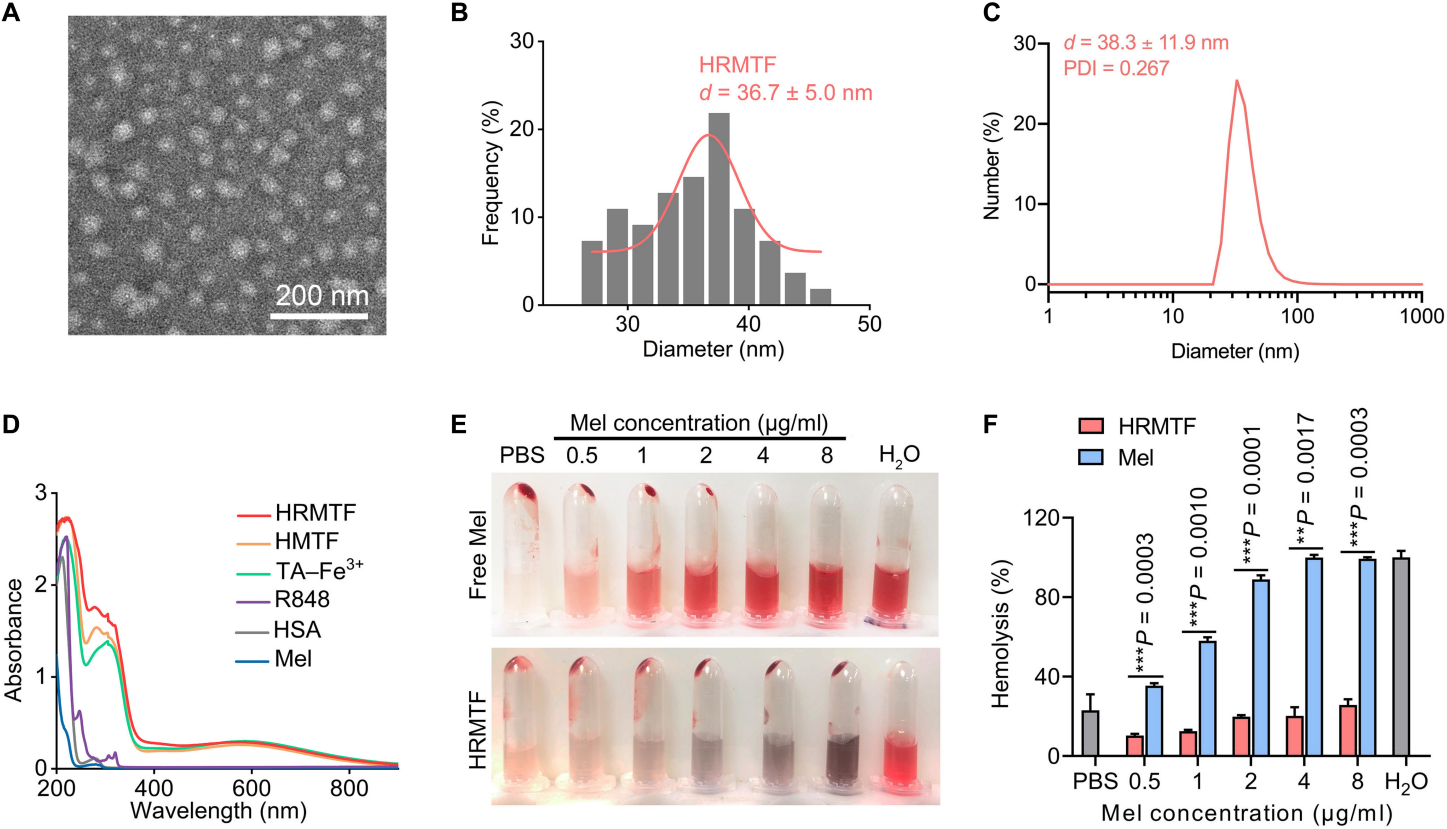
(A) TEM picture, (B) corresponding size distribution result, and (C) hydrodynamic diameter of HRMTF NPs. (D) UV–vis absorption spectra of solutions/suspensions of Mel, HSA, R848, TA–Fe^3+^, HMTF, or HRMTF. (E) Pictures of different concentrations of Mel- or HRMTF-treated RBC suspensions. The RBC suspensions treated with H_2_O and phosphate-buffered saline (PBS) were used as positive and negative control, respectively. (F) Corresponding hemolysis rates of the RBC samples in (E). **P < 0.01, ***P < 0.001.

The HRMTF NPs were fabricated according to the procedures depicted in Fig. 1A. Briefly, Mel and HSA solutions were mixed and added with R848 solution under stirring. Then, the solution of TA was added to the above mixed solution for crosslinking the proteins and stabilizing the Mel, because the dihydroxyphenyl and trihydroxyphenyl groups in the TA molecules can interact with the R848-encapsulated HSA–Mel protein/peptide mixture via noncovalent interactions [17,50]. Finally, Fe^3+^ was added to coordinate with TA and form the TA–Fe^3+^ MPNs. Fe^3+^ could weaken the strong forces between the peptide/protein mixture and TA and stabilize the HRMTF NPs. By using dynamic light scattering (DLS) to study the hydrodynamic sizes of the formed NPs, the optimal mass ratio of HSA:R848:Mel:TA:Fe^3+^ (*m*(HSA):*m*(R848):*m*(Mel):*m*(TA):*m*(Fe^3+^)) was determined to be 100:1.6:20:14.8:4.14. The transmission electron microscopy (TEM) image showed that the fabricated HRMTF NPs had a uniform spherical structure with a mean diameter of ~36.7 nm (Fig. 2A and B), and the DLS analysis illustrated that the hydrodynamic diameter of the HRMTF NPs was ~38.3 nm (Fig. 2C). Besides, the zeta potential value of HRMTF was −16.0 mV (Fig. S1), which was due to the carboxyl groups of TA. The ultraviolet–visible (UV–vis) absorption spectra also confirmed the successful fabrication of the HRMTF NPs (Fig. 2D). Then, HSA–TA–Fe^3+^ (HTF), HSA–R848–TA–Fe^3+^ (HRTF), and HSA–Mel–TA–Fe^3+^ (HMTF) NPs were also constructed similarly for comparison purposes, and the TEM images and the DLS results illustrated the successful fabrication of these 3 kinds of NPs (Figs. S2 and S3). Then, the formation mechanisms of the HRMTF NPs were investigated by separately adding urea, Triton X-100, NaCl, and ethylenediaminetetraacetic acid disodium salt (EDTA) solutions into the HRMTF suspensions to disrupt the hydrogen bonds, hydrophobic interactions, electrostatic attraction interactions, and coordination bonds within the NPs, respectively. The DLS analyses of the HRMTF NPs after various treatments were carried out. As illustrated in Fig. S4, the hydrodynamic diameter of the NaCl-treated HRMTF NPs was similar to that of the untreated ones, indicating that the electrostatic attraction interaction was not the major formation force of the HRMTF NPs. On the other hand, the hydrodynamic diameters of the urea-, Triton X-100-, and EDTA-treated HRMTF NPs were obviously different from that of the untreated ones, indicating that the hydrogen bonding, hydrophobic interaction, and coordination bonding play a major role in the formation of the HRMTF NPs.

To check if the hemolytic activity of Mel could be shielded by the HRMTF NPs, we measured the hemolysis rate of the HRMTF NPs. Compared with free Mel molecules, the hemolysis ability of HRMTF NPs was drastically declined (Fig. 2E and F). Specifically, negligible hemolysis was observed in the HRMTF-treated red blood cell (RBC) suspensions even at a high Mel concentration of 8 μg/ml, suggesting that the hemolytic activity of Mel encapsulated within the HRMTF NPs was largely suppressed, which was beneficial for ensuring the good in vivo safety of the HRMTF NPs.

### Cellular internalization and anticancer efficacy of HRMTF NPs in vitro

**Fig. 3.**
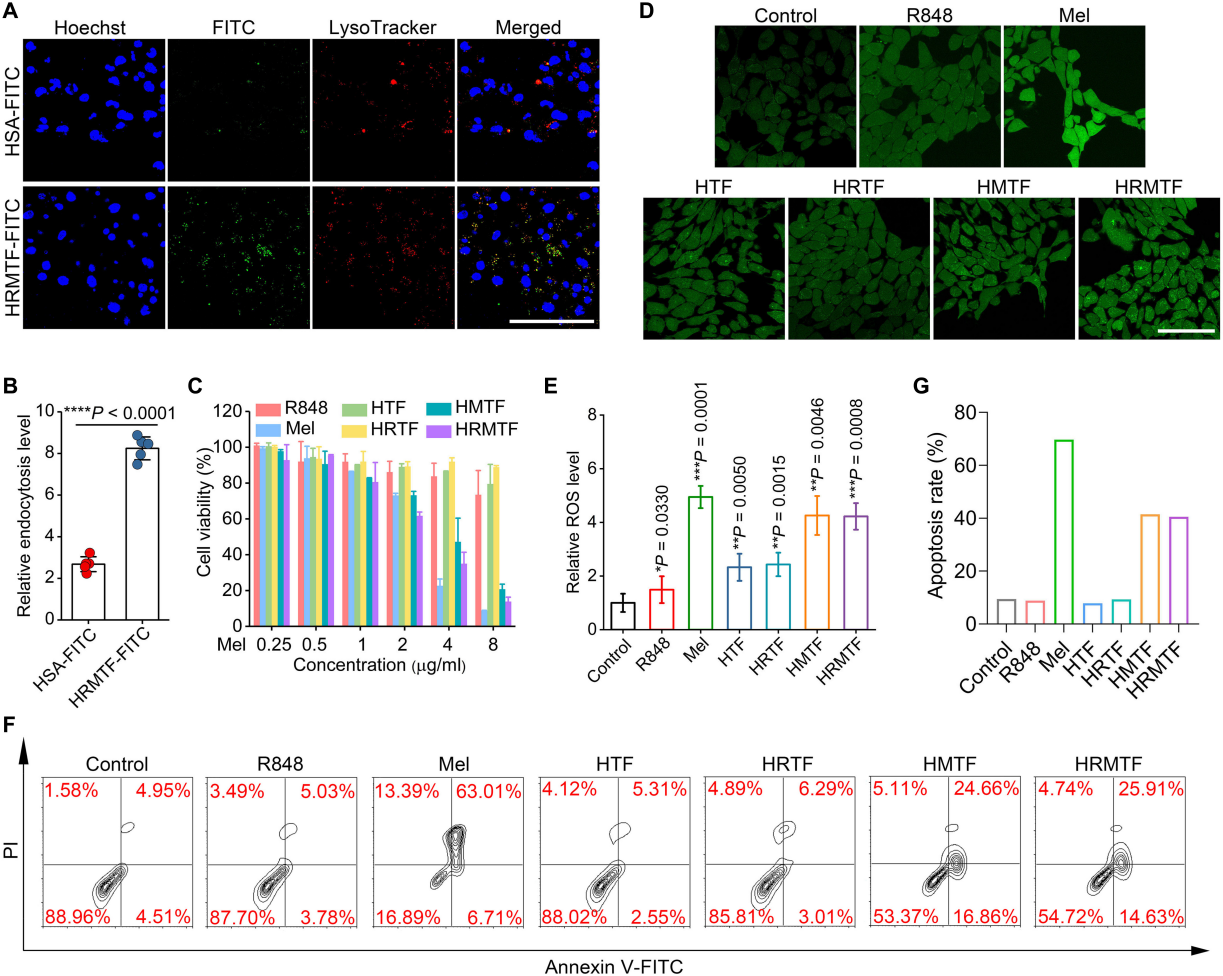
(A) Confocal imaging results of 4T1 tumor cells after treated with HSA-FITC or HRMTF-FITC for 2 h. The cell nuclei were labeled with Hoechst 33342 (Hoechst) and the lysosomes were labeled with LysoTracker Red (LysoTracker) before imaging. Scale bar: 75 μm. (B) Endocytosis levels of the 4T1 cells subjected to HSA-FITC or HRMTF-FITC treatment. The levels were derived from the flow cytometric results in Fig. S5. ****P < 0.0001. (C) Relative viabilities of 4T1 cells after incubation with different doses of R848, Mel, HTF, HRTF, HMTF, and HRMTF, respectively. (D) Confocal imaging and (E) flow cytometric results showing the ROS levels (reflected by DCFH-DA) in 4T1 cells after various treatments as illustrated. *P < 0.05, **P < 0.01, ***P < 0.001. Flow cytometric results (F) and corresponding apoptosis rates (G) of 4T1 cells after various treatments as indicated. For (A), (B), and (D) to (G), the Mel concentration was 4 μg/ml.

Next, to investigate the cellular internalization of HRMTF NPs, the fluorescein isothiocyanate (FITC)-linked HSA (HSA-FITC) was used to prepare FITC-labeled HRMTF (termed HRMTF-FITC) NPs. The confocal imaging and flow cytometric results illustrated that the cellular internalization amount of the HRMTF-FITC NPs by murine mammary carcinoma cells (4T1 cells) was much higher than that of HSA-FITC (Fig. 3A and B and Fig. S5). Moreover, the confocal imaging results also revealed that the fluorescence of HRMTF-FITC NPs (green) did not overlap with the fluorescence of LysoTracker Red (red, a commercial lysosomal staining dye) (Fig. 3A), indicating that the HRMTF-FITC NPs could evade the capture of lysosomes after internalization.

Then, the in vitro anticancer performance of the HRMTF NPs was studied using 4T1 cells. Not surprisingly, free Mel showed the highest toxicity to 4T1 cells (Fig. 3C), which was attributed to its efficient membrane-disrupting ability. Besides, HMTF and HRMTF also showed high anticancer efficacies (Fig. 3C), reflecting the combinational effect of the Mel-mediated cell membrane lysis and the TA–Fe^3+^ MPN-induced Fenton reaction. In contrast, R848, HTF, and HRTF showed negligible cytotoxicity, indicating that the TA–Fe^3+^ alone was not efficient for inducing cell death and the R848 elicited very low toxicity to cancer cells. Since the coordination force between TA and Fe^3+^ became weaker under an acidic environment, we wondered if the tumor microenvironment (pH ≈ 6.5) and the acidic endo/lysosome environment (pH ≈ 5.5) in the tumor cells would facilitate the disassembly of the HRMTF NPs and enhance their cytotoxicity. Therefore, the HRMTF NPs dispersed in solutions with a pH value of 6.5 and 5.5 were characterized by TEM and DLS (Figs. S6 and S7). The results demonstrated that the diameter of the HRMTF NPs increased to ~63 nm at pH 6.5, indicating that the HRMTF NPs were partially influenced in the tumor microenvironment. Moreover, the HRMTF NPs were turned into small particles (~23 nm, as revealed by the TEM data), indicating that the HRMTF NPs were disassembled in the endo/lysosomes after internalization.

The TA–Fe^3+^-induced Fenton reaction could produce the highly toxic HO• in the presence of H_2_O_2_, which was overexpressed in the tumor cells. Thus, the HO•-producing capacity of the HTF NPs in the presence of H_2_O_2_ was analyzed via measuring the absorbance of methylene blue (MB), which could be faded by HO•. The results shown in Fig. S8 revealed that the HTF NPs displayed robust HO• generation ability after being mixed with H_2_O_2_, demonstrating their superior Fenton-reaction-inducing ability. Because the Mel-mediated membrane disruption and TA–Fe^3+^-induced Fenton reaction might both produce reactive oxygen species (ROS) for cell killing, we next studied the ROS-producing capacity of various samples in 4T1 tumor cells using the ROS probe 2′,7′-dichlorodihydrofluorescein diacetate (DCFH-DA). There was a positive correlation between green fluorescence intensity (from 2′,7′-dichlorofluorescein [DCF] that was formed by oxidizing the 2′,7′-dichlorodihydrofluorescein [DCFH], which was the hydrolysis production of DCFH-DA) and intracellular ROS content. The brightest fluorescence was observed in the Mel- and HRMTF-treated 4T1 cells (Fig. 3D and E and Fig. S9), indicating the strong ROS-producing ability of Mel and HRMTF NPs. Besides, the Mel-, HMTF-, and HRMTF-treated 4T1 cells also showed high apoptosis rates (Fig. 3F and G), which was consistent with the cytotoxicity results.

### In vitro ICD effect and immunostimulation effect of HRMTF NPs

**Fig. 4.**
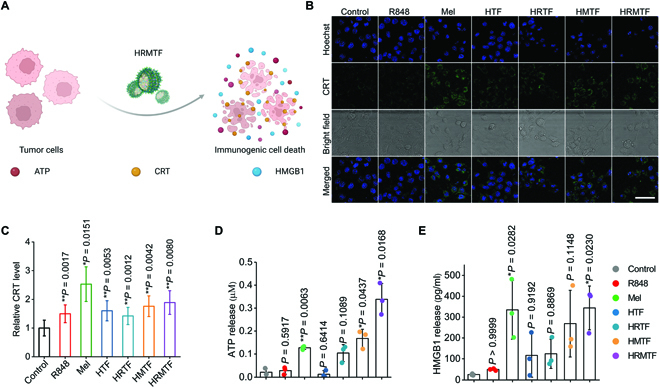
(A) Scheme illustrating the ICD-induced DAMP (e.g., CRT, ATP, and HMGB1) release/exposure from dying tumor cells. (B) Confocal imaging results and (C) flow cytometric results of CRT levels on 4T1 cells after various treatments. Scale bar: 50 μm. Quantification of extracellular levels of (D) ATP and (E) HMGB1 after various treatments. The Mel content in all samples was fixed at 4 μg/ml. *P < 0.05, **P < 0.01, ***P < 0.001.

During the HRMTF-induced cell death process, Mel could interact with plasma membrane and induce cell lysis, and TA–Fe^3+^-induced Fenton reaction could produce ROS. Thus, we wondered if the HRMTF-derived cell death belonged to ICD and could induce the release/exposure of DAMPs. To this end, the release/exposure of 3 vital DAMPs, i.e., adenosine triphosphate (ATP), calreticulin (CRT), and high-mobility group box 1 (HMGB1) protein, were analyzed to evaluate the HRMTF-induced ICD effect (Fig. 4A). The results indicated that the R848-, Mel-, HTF-, HRTF-, HMTF-, and HRMTF-treated 4T1 cells all had a higher CRT exposure level on the cell membrane than the control group (Fig. 4B and C and Fig. S10), indicating the ICD effect of these drugs. Next, the ATP release and HMGB1 secretion were also studied by a luciferase-based ATP assay and the enzyme-linked immunosorbent assay (ELISA), respectively. Compared with other groups, the HRMTF-treated cells exhibited the highest ATP release and HMGB1 secretion levels (Fig. 4D and E), indicating the strong ICD-inducing effect of HRMTF NPs. Collectively, the HRMTF NPs could induce efficient ICD of 4T1 cells by enhancing the release/exposure of DAMPs.

**Fig. 5.**
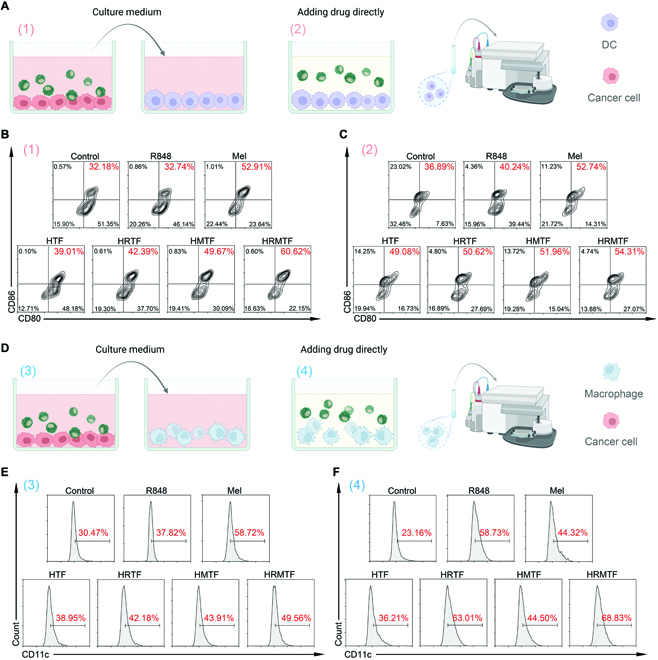
(A) Schematic illustration of DAMP (1)- and drug (2)-induced BMDC maturation in vitro. (B and C) Flow cytometric results of mature DCs (CD11c^+^CD80^+^CD86^+^) after incubation with the culture media of the 4T1 cells after different treatments as indicated (B) or the culture media containing different drugs as indicated (C). (D) Schematic illustration of DAMP (3)- and drug (4)-induced M1-like repolarization of macrophages in vitro. (E and F) Flow cytometric results of M1-like macrophages (CD11c^+^F4/80^+^) after incubation with the culture media of the 4T1 cells after different treatments as indicated (E) or the culture media containing different drugs as indicated (F).

Since the HRMTF-treated tumor cells would undergo ICD and secrete/expose “eat me signals” (i.e., DAMPs), we wondered if these DAMPs together with the tumor-associated antigens could be presented to immature dendritic cells (DCs) and M2-like macrophages for DC maturation and M1-like macrophage repolarization and finally stimulate the immune system for cancer immune therapy. Thus, the immunostimulation effect of the HRMTF-treated 4T1 cells were studied [Fig. 5A and D, (1) and (3)].

First, the maturation of DCs and the M1-like repolarization of macrophages were investigated using the bone marrow-derived DCs (BMDCs) and RAW 264.7 cells (mouse macrophages), respectively. Briefly, 4T1 cells were treated with culture medium (control), R848, Mel, HTF, HRTF, HMTF, or HRMTF for 4 h, and the culture media were replaced by the fresh ones and incubated for 20 h. Then, the culture media containing the “secretome” of various drug-treated 4T1 cells were used to incubate immature DCs and M2-like macrophages for 24 h, and the DC maturation and M1-like repolarization were analyzed by flow cytometry. The results illustrated that the highest rate of mature BMDCs (CD11c^+^CD80^+^CD86^+^) (60.62%) was detected in the HRMTF group (Fig. 5B and Fig. S11), indicating that the HRMTF-induced ICD could stimulate the immature DCs to form mature DCs. Besides, a large proportion of M1-like macrophages (F4/80^+^CD11c^+^) (49.56%) was observed in the HRMTF group, which was lower than that of the Mel group (58.72%) (Fig. 5E and Fig. S12), demonstrating that the Mel-induced cell membrane lysis could induce the M1-like repolarization of macrophages, and this ability of Mel was partially retained after being loaded in the HRMTF NPs.

On the other hand, considering that R848 can act as an immune adjuvant for immunostimulation [51–53], and the low content of ROS, which could be produced by the TA–Fe^3+^ in the HRMTF NPs, could also induce the M1-like repolarization of macrophages [54], we also treated immature DCs and M2-like macrophages directly with various samples to evaluate the immunostimulation outcomes [Fig. 5A and D, (2) and (4)]. Not surprisingly, the HRMTF-treated BMDCs showed the highest maturation proportion (54.31%) among all the groups (Fig. 5C), and the HRMTF NPs could also facilitate the M1-like repolarization of macrophages (68.83%) (Fig. 5F). It is worth mentioning that R848 was important for the M1-like repolarization of macrophages, since the R848-containing drugs (e.g., R848, HRTF, and HRMTF) all had a strong M1-like macrophage repolarization-inducing ability (Fig. 5F).

Collectively, the HRMTF NPs could not only induce ICD of cancer cells, but also directly interact with immature DCs or M2-like macrophages for their maturation or M1-like repolarization.

### In vivo anticancer performance and abscopal effect of HRMTF NPs

**Fig. 6.**
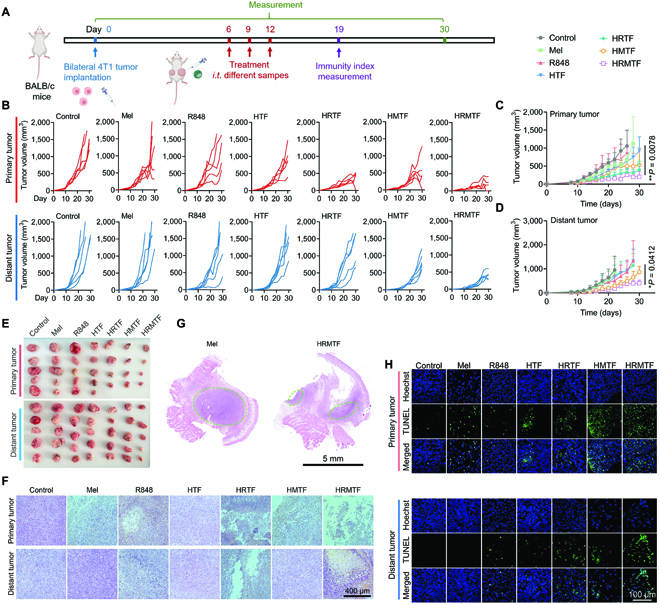
(A) Experimental outline showing the steps for evaluating the therapeutic and immunostimulation outcomes of various drugs in the bilateral 4T1 tumor-bearing BALB/c mice. (B) Individual primary and distant 4T1 tumor growth curves after various treatments (*n* = 5/group). (C and D) Average primary (C) and distant (D) tumor growth curves of the bilateral 4T1 tumor-bearing BALB/c mice after different treatments (*n* = 5/group). *P < 0.05, **P < 0.01, based on the comparison between the control group and the HRMTF group. (E) Photographs of primary and distant 4T1 tumors after different treatments (*n* = 5/group). (F) Confocal microscopic images of representative H&E staining results of tumor slices from the primary and distant 4T1 tumors of the bilateral 4T1 tumor-bearing BALB/c mice after various treatments. Scale bar: 400 μm. (G) Scanning photos of the primary tumor tissue slices from the bilateral 4T1 tumor-bearing BALB/c mice after Mel or HRMTF treatment. Scale bar: 5 mm. (H) Confocal microscopic images of representative TUNEL assay results of the primary and distant tumor tissue slices from the bilateral 4T1 tumor-bearing BALB/c mice after various treatments. Scale bar: 100 μm.

Encouraged by the superb ICD-inducing and immunostimulation effects of HRMTF NPs in vitro, we further investigated their in vivo cancer therapeutic effect using the bilateral 4T1 tumor-bearing BALB/c mouse models. The right flank of the mice was implanted with 4T1 tumor cells to establish the primary tumor and the left flank was inoculated with 5 times fewer cells to form the distant tumor. Six days later, the above mice were indiscriminately divided into 7 groups and intratumorally injected 3 times with PBS (control), Mel, R848, HTF, HRTF, or HRMTF in the primary tumor every 3 days (Fig. 6A). Notably, the strongest primary tumor suppression was observed in the HRMTF-injected mice (Fig. 6B, C, and E). Besides, the HMTF and HRTF could also partially suppress the primary tumor, while the primary tumor inhibition was not satisfactory enough in the Mel-treated mice, where only 2 out of the 5 primary tumors were notably suppressed. Importantly, the growth of the distant tumors of the HRMTF-treated mice was also considerably slowed (Fig. 6B, D, and E), possibly owing to the ICD of the tumor cells in the primary tumors and the immunostimulation effect of the released R848. The distant tumors in the HRTF and HMTF groups were also inhibited, but the inhibition outcomes were not as evident as that in the HRMTF group.

Moreover, the hematoxylin and eosin (H&E)-stained tumor tissue slices also revealed that both the primary and distant tumors of the HRMTF-treated mice were severely destroyed (Fig. 6F). In addition, the entire H&E-stained primary tumor tissue slices of the Mel- and HRMTF-treated mice indicated that the Mel could only destroy the peripheral tumor tissue, leaving the deep tumor tissue unaffected, while the HRMTF NPs could ruin most areas of the tumor tissue (Fig. 6G). Thus, the unsatisfactory primary tumor suppression outcome of Mel might be attributed to its poor tumor-penetrating efficiency. Besides, free Mel could hardly inhibit the growth of the distant tumors, indicating that Mel was not enough to arouse efficient antitumor immunostimulation. Moreover, strong green fluorescence signals were detected in both tumors of the HRMTF-treated mice after terminal deoxynucleotidyl transferase (TdT)-mediated dUTP nick end labeling (TUNEL) assay kit staining (Fig. 6H), indicating the high level of cell apoptosis in the HRMTF-treated tumor tissues. Next, the ROS-producing ability of HTF NPs, HSA-R848 + Mel mixture, and HRMTF NPs in the primary tumor was evaluated. The results indicated that both HTF NPs and HSA-R848 + Mel mixture could produce ROS (red) in the tumor tissues (Fig. S13), and their combination effect was proved by the enhanced red fluorescence in the HRMTF-treated tumor tissue, which indicated the strongest ROS-producing ability of the HRMTF NPs. Collectively, the above results strongly proved the superb antitumor effect of HRMTF NPs in vivo.

### Intratumoral and systemic antitumor immunostimulation effects of HRMTF NPs

**Fig. 7.**
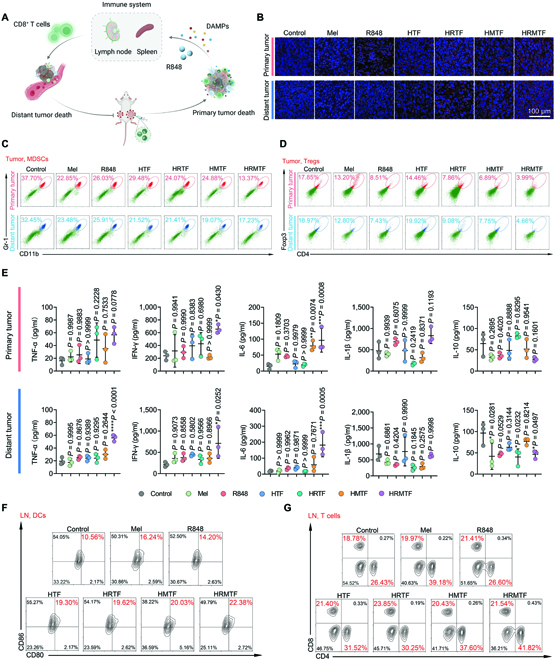
(A) Scheme of the HRMTF-induced intratumoral and systemic anticancer immunostimulation in vivo. (B) Confocal fluorescence images of the immunofluorescence staining results of CRT of the tumor tissue slices from the bilateral 4T1 tumor-bearing BALB/c mice taken at day 7 after various treatments. Scale bar: 100 μm. (C and D) Representative flow cytometric plots of MDSCs (CD11b^+^Gr-1^+^) (C) and Tregs (CD3^+^CD4^+^Foxp3^+^) (D) in the tumor tissues retrieved from the bilateral 4T1 tumor-bearing BALB/c mice 7 days after various treatments. (E) Intratumoral contents of TNF-α, IFN-γ, IL-6, IL-1β, and IL-10 analyzed by ELISA (*n* = 3/group). *P < 0.05, **P < 0.01, ***P < 0.001, ****P < 0.0001. (F and G) Representative flow cytometric plots of DCs (CD11c^+^CD80^+^CD86^+^) (F), helper T cells (CD3^+^CD4^+^), and cytotoxic T cells (CD3^+^CD8^+^) (G) in the LNs retrieved from the bilateral 4T1 tumor-bearing BALB/c mice 7 days after various treatments.

Owing to the superb in vitro ICD-inducing and immunostimulation effects and the impressive distant tumor inhibition outcome of HRMTF NPs, we wondered if the HRMTF NPs could induce the intratumoral and systemic antitumor immunostimulation for cancer immune therapy. Therefore, the intratumoral and systemic immunostimulation abilities of various drugs were further evaluated (Fig. 7A).

As illustrated by the confocal fluorescence images, the most CRT antibody-stained red fluorescence signals were detected in the HRMTF-injected primary tumor tissues (Fig. 7B), indicating the strongest in vivo ICD-inducing effect of the HRMTF NPs. Then, the immunocellular profiles of the tumor tissues in different groups were studied. The flow cytometric results showed that the HRMTF NPs could remarkably increase the proportion of the mature DCs (CD11c^+^CD80^+^CD86^+^) both in the primary tumor (39.01%) and in the distant tumor (41.77%) relative to that in the control group (primary tumor: 15.76%; distant tumor: 20.70%) (Fig. S14). Besides, the proportion of the intratumoral M1-like macrophages (F4/80^+^CD11c^+^) was also higher in the HRMTF group (primary tumor: 28.21%; distant tumor: 34.76%) than that of the control group (primary tumor: 6.36%; distant tumor: 10.82%) (Fig. S15). Meanwhile, the intensity of red fluorescence signals of CD206 was decreased in the tumor tissues after HRMTF treatment (Fig. S16), demonstrating the decreased intratumoral number of M2-like macrophages in the HRMTF-treated tumor tissues. Moreover, after being treated with the HRMTF NPs, the intratumoral helper T cells (CD3^+^CD4^+^) and cytotoxic T cells (CD3^+^CD8^+^) were both largely increased relative to those in the control group (Fig. S17), demonstrating that the HRMTF NPs could finally stimulate the local immune system for cancer therapy. By contrast, the free Mel could only activate the T cells in the primary tumor, but failed to boost the abscopal effect (Fig. S17). In addition to the above-mentioned M2-like macrophages, 2 other kinds of immunosuppressive cells (i.e., myeloid-derived suppressor cells [MDSCs] and regulatory T cells [Tregs]) in the tumor tissues were also evaluated. Not surprisingly, the HRMTF treatment could considerably decrease the intratumoral proportions of MDSCs (Gr-1^+^CD11b^+^) and Tregs (CD3^+^CD4^+^Foxp3^+^) both in the primary tumors (13.37% for MDSCs and 3.99% for Tregs) and in the distant tumors (17.23% for MDSCs and 4.66% for Tregs) (Fig. 7C and D and Figs. S18 and S19), further indicating the intratumoral immunostimulation effect of the HRMTF NPs. As for the intratumoral levels of some representative cytokines, the ELISA results showed that the intratumoral contents of interferon-γ (IFN-γ), tumor necrosis factor-α (TNF-*α*), and interleukin-6 (IL-6), which are the cytokines associated with innate immunostimulation, were largely elevated in both the primary and distant tumors, while the concentration of the anti-inflammatory interleukin-10 (IL-10) was around the baseline level in the primary tumors and reduced in the distant tumors of the HRMTF-treated mice (Fig. 7E). In general, the above results demonstrated that the HRMTF NPs could substantially arouse the intratumor anticancer immune responses and boost the abscopal effect.

To figure out the mechanism behind the potentiated distant tumor inhibition ability of the HRMTF NPs, we further analyzed the systemic anticancer immunostimulation effect of various drugs. The cells of the spleens and lymph nodes (LNs) from the treated mice were isolated and analyzed. The results illustrated that the highest mature DC (CD11c^+^CD80^+^CD86^+^) level was observed in the spleens (34.18%) and LNs (22.38%) of the HRMTF-treated mice (Fig. 7F and Figs. S20 and S21A). In addition, the HRMTF NPs could also increase the rates of M1-like macrophages (F4/80^+^CD11c^+^) in spleens (22.74%) and LNs (17.07%) relative to those in the control group (Figs. S22 and S23). Importantly, the helper T cells (CD3^+^CD4^+^) and cytotoxic T cells (CD3^+^CD8^+^) were both considerably enriched in the spleens and LNs after HRMTF treatment (Fig. 7G and Figs. S21B and C, S24, and S25). Specifically, the proportions of these 2 kinds of T cells in the spleens of the HRMTF-treated mice were both approximately 3-fold higher than those in the control group (Fig. S25). Furthermore, the serum contents of the proinflammatory TNF-α and IFN-γ were largely increased while the antiinflammatory IL-10 was almost unchanged after HRMTF treatment (Fig. S26), indicating the systemic antitumor immunostimulation effect of HRMTF NPs.

Collectively, the above results indicated that the HRMTF NPs could not only cause the ICD of tumor cells and facilitate the intratumoral immune cell recruitment and stimulation, but also arouse the systemic immunostimulation to suppress the growth of the distant tumors via the abscopal effect.

### In vivo biocompatibility of HRMTF NPs

**Fig. 8.**
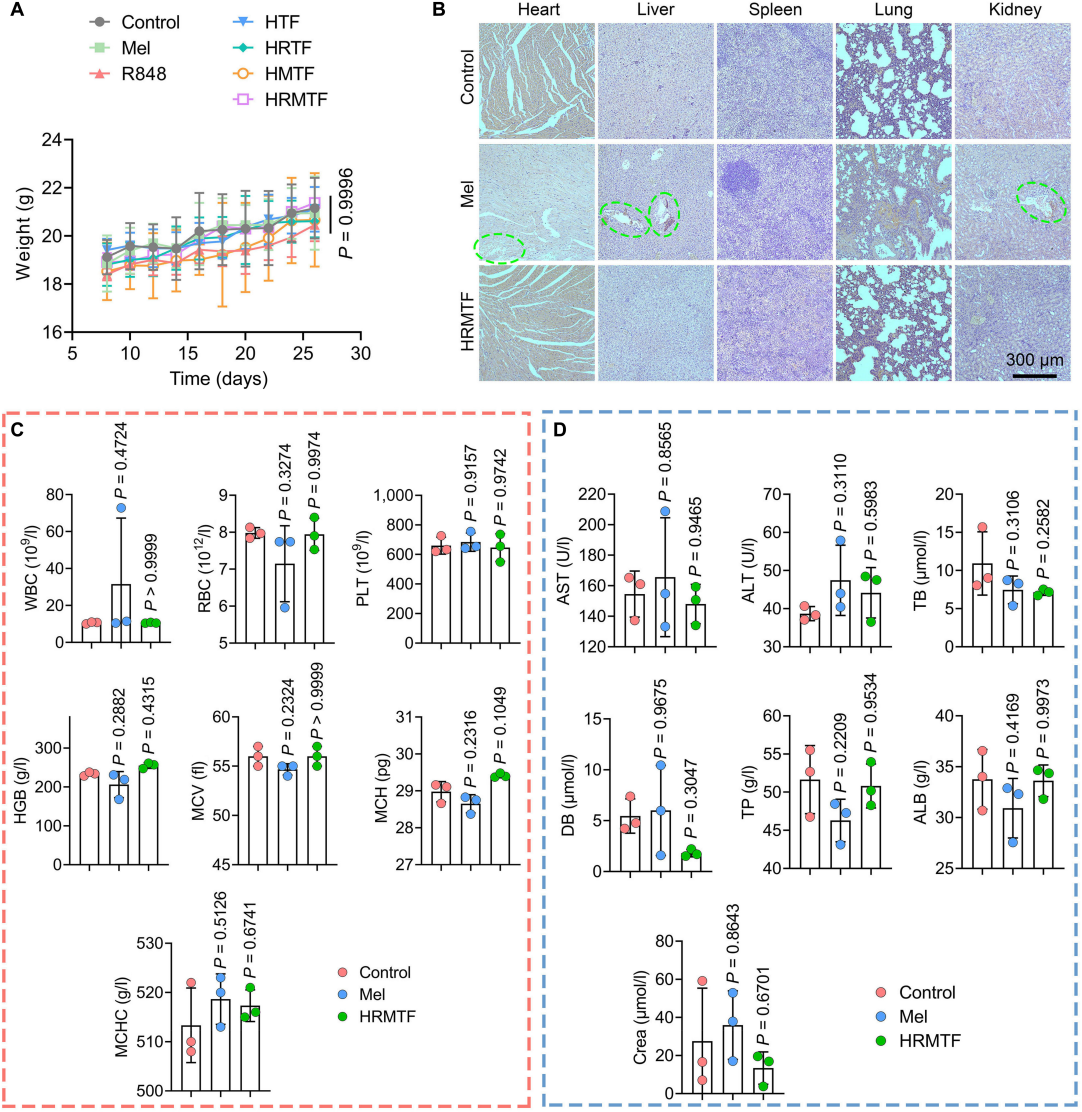
(A) Body weight changes of the bilateral 4T1 tumor-bearing BALB/c mice after various treatments. (B) H&E-stained tissue slices of the major organs as indicated excised from the BALB/c mice on day 14 after PBS (control), Mel, or HRMTF treatment (Mel dose: 5 mg/kg). Scale bar: 300 μm. (C) Routine blood analysis results of the BALB/c mice measured on day 14 after PBS (control), Mel, or HRMTF treatment (Mel dose: 5 mg/kg; *n* = 3/group). The blood indexes including WBC, RBC, PLT, HGB, MCV, MCH, and MCHC indicate the numbers of white blood cells, red blood cells, and platelets, concentration of hemoglobin, mean corpuscular volume, mean corpuscular hemoglobin, and mean corpuscular hemoglobin concentration, respectively. (D) Biochemical analysis results of the blood samples measured from the BALB/c mice on day 14 after PBS (control), Mel, or HRMTF treatment (Mel dose: 5 mg/kg; *n* = 3/group). The corresponding indexes include aspartate transferase (AST), alanine transferase (ALT), total bilirubin (TB), direct bilirubin (DB), total protein (TP), albumin (ALB), and creatinine (Crea).

Finally, the in vivo biocompatibility and off-target toxicity of the HRMTF NPs were evaluated. First, almost no body weight loss was detected during the experimental period in all the groups (Fig. 8A). The H&E staining results illustrated that no abnormal tissues were observed in the HRMTF group (Fig. 8B). In contrast, some apparent tissue damage in the heart, liver, and kidney was observed in the Mel group (Fig. 8B), revealing the strong off-target toxicity of free Mel. Moreover, all the tested indexes in the routine blood and biochemical analyses of the HRMTF-treated mice were similar to those in the control group (Fig. 8C and D), indicating the good hemocompatibility of the HRMTF NPs. In general, the HRMTF NPs had satisfactory biocompatibility, ensuring their potential clinical translation.

## Conclusion

In conclusion, we reported the facile construction of an MPN-based drug delivery nanosystem containing Mel and an immune adjuvant (R848) for cancer immunotherapy. The fabricated HRMTF NPs could convert Mel, which has a strong hemolytic activity due to its indiscriminate membrane-disrupting property, from foe to friend and exhibited good biocompatibility toward the blood and normal tissues. Besides, we have confirmed that the hemolytic activity of Mel was shielded by the HRMTF NPs to avoid the risk of the possible leakage of the HRMTF NPs in blood after intratumoral injection and ensure their in vivo safety. In the tumor regions, the combination of the Mel-induced membrane disruption and the MPN-mediated Fenton reaction-produced ROS resulted in efficient ICD and irreversible damage of the tumor tissues. Moreover, the HRMTF NPs could efficiently recruit immune cells into the tumor tissues, activate the antigen-presenting cells (DCs), and arouse robust intratumoral and systemic antitumor immunostimulation. In the bilateral 4T1 tumor-bearing BALB/c mouse models, the HRMTF NPs were able to considerably damage the treated primary tumors and also boosted the abscopal effect to suppress the growth of the distant tumors.

Additionally, it is expected that the MPN-based drug delivery nanosystem can also be used to load other kinds of functional proteins and immune agonists to further amplify the cancer therapeutic effect in the future. Immune checkpoint blockade (ICB) therapy can also be combined with this nanosystem to further improve the immunostimulation and abscopal effects. The frame of the nanosystem (i.e., MPN and HSA) has been extensively proved to be biocompatible and the payloads (i.e., R848 and Mel) have also been widely studied, which facilitate the possible clinical applications of the HRMTF nanosystem. Finally, the MPN-mediated “foe-to-friend” strategy may inspire the development of more efficacious nanodrugs for diverse applications.

## Methods

### Preparation of HRMTF

First, 1 ml of HSA suspension (2 mg/ml, in H_2_O) and 40 μl of Mel solution (10 mg/ml, in H_2_O) were mixed together and stirred, followed by adding 10 μl of R848 solution (10 mM, in DMSO). The obtained mixture was stirred, followed by adding 200 μl of TA solution (2 mg/ml, in H_2_O) upon shaking. Finally, 200 μl of FeCl_3_ solution (10 mM, in H_2_O) was introduced to the above mixture and the resultant dark blue suspension was vortexed for 30 s, followed by centrifugation (8,000 rpm, 5 min) to remove the excess Fe^3+^–TA MPNs and R848 molecules. The resultant suspension was vortexed for 10 s to obtain the final HRMTF NPs with an *m*(HSA):*m*(R848):*m*(Mel):*m*(TA):*m*(Fe^3+^) ratio of 100:1.6:20:14.8:4.14 (determined by UV–vis spectroscopy using a Shimadzu UV-2600 spectrophotometer, Japan). HTF, HRTF, and HMTF were prepared in a similar way.

### Characterization

The size and morphology of the HTF, HRTF, HMTF, and HRMTF NPs were characterized by a transmission electron microscope (JEM-2100, JEOL Ltd., Japan). The hydrodynamic sizes and zeta potentials of various NPs were measured by a zetasizer instrument (Nano ZS, Malvern Instruments, UK). The UV–vis absorption spectra of Mel, HSA, R848, TA–Fe^3+^, HMTF, and HRMTF solutions/suspensions were recorded using a Shimadzu UV-2600 spectrophotometer (Japan).

## Data Availability

The data that support the findings of this study are available within the article and its supplementary materials. Raw data are available from the corresponding authors on reasonable request.

## Supplementary Materials

Supplementary Materials Experimental section. Supplementary figures. Fig. S1. Zeta potential of HRMTF NPs. Fig. S2. TEM images and corresponding size distribution results of HTF, HRTF, and HMTF NPs. Fig. S3. Hydrodynamic diameters of HTF, HRTF, and HMTF NPs. Fig. S4. Hydrodynamic diameters of HRMTF NPs after the treatment of Triton X-100, NaCl, EDTA, or urea. Fig. S5. Representative flow cytometric plots of the FITC fluorescence intensities in the 4T1 cells after treatment with HSA-FITC or HRMTF-FITC. Fig. S6. TEM images and corresponding size distribution results of HRMTF NPs at pH 7.4, 6.5, and 5.5. Fig. S7. Hydrodynamic diameters of HRMTF NPs at pH 7.4, 6.5, and 5.5. Fig. S8. UV–vis absorption spectra of the solutions containing MB + HTF or MB + HTF + H_2_O_2_. Fig. S9. Representative flow cytometric plots of the ROS levels in the 4T1 cells after various treatments. Fig. S10. Representative flow cytometric plots of the ROS levels in the 4T1 cells after various treatments. Fig. S11. Representative flow cytometry gating strategy for mature DCs. Fig. S12. Representative flow cytometry gating strategy for M1-like macrophages. Fig. S13. Confocal microscopic images of ROS-staining results of the tumor tissue slices after various treatments. Fig. S14. Representative flow cytometric plots and corresponding quantification results of DCs in the tumor tissues retrieved from mice after various treatments. Fig. S15. Representative flow cytometric plots and corresponding quantification results of M1-like macrophages in the tumor tissues retrieved from mice after various treatments. Fig. S16. Confocal fluorescence images of the immunofluorescence staining results of CD206 of the tumor tissue slices retrieved from mice after various treatments. Fig. S17. Confocal fluorescence images of the immunofluorescence staining results of CD4 and CD8 of the tumor tissue slices retrieved from mice in different groups. Fig. S18. Quantification of the flow cytometric results of MDSCs in the tumor tissues retrieved from mice in different groups. Fig. S19. Quantification of the flow cytometric results of Tregs in the tumor tissues retrieved from mice in different groups. Fig. S20. Representative flow cytometric plots and corresponding quantification results of mature DCs in the spleens retrieved from mice in different groups. Fig. S21. Quantification of the flow cytometric results of mature DCs, helper T cells, and cytotoxic T cells in the LNs retrieved from mice after various treatments. Fig. S22. Representative flow cytometric plots and corresponding quantification results of M1-like macrophages in the spleens retrieved from mice after various treatments. Fig. S23. Representative flow cytometric plots and corresponding quantification results of M1-like macrophages in the LNs retrieved from mice after various treatments. Fig. S24. Representative flow cytometry gating strategy for CD3^+^CD4^+^ or CD3^+^CD8^+^ T cells. Fig. S25. Representative flow cytometric plots and corresponding quantification results of helper T cells and cytotoxic T cells in the spleens retrieved from mice after various treatments. Fig. S26. Expression levels of TNF-α, IFN-γ, and IL-10 in the serum retrieved from mice after various treatments. (Supplementary Materials)
