# Supplementary material for: Metal–Phenolic Network-Facilitated “Foe-to-Friend” Conversion of Melittin for Cancer Immunotherapy with Boosted Abscopal Effect
Source: Research (Wash D C). 2023 Mar 8;6:0052. doi: 10.34133/research.0052 (PMC10013787; doi:10.34133/research.0052)
Supplement: Supplementary Materials — Experimental section. Supplementary figures. Fig. S1. Zeta potential of HRMTF NPs. Fig. S2. TEM images and corresponding size distribution results of HTF, HRTF, and HMTF NPs. Fig. S3. Hydrodynamic diameters of HTF, HRTF, and HMTF NPs. Fig. S4. Hydrodynamic diameters of HRMTF NPs after the treatment of Triton X-100, NaCl, EDTA, or urea. Fig. S5. Representative flow cytometric plots of the FITC fluorescence intensities in the 4T1 cells after treatment with HSA-FITC or HRMTF-FITC. Fig. S6. TEM images and corresponding size distribution results of HRMTF NPs at pH 7.4, 6.5, and 5.5. Fig. S7. Hydrodynamic diameters of HRMTF NPs at pH 7.4, 6.5, and 5.5. Fig. S8. UV–vis absorption spectra of the solutions containing MB + HTF or MB + HTF + H2O2. Fig. S9. Representative flow cytometric plots of the ROS levels in the 4T1 cells after various treatments. Fig. S10. Representative flow cytometric plots of the ROS levels in the 4T1 cells after various treatments. Fig. S11. Representative flow cytometry gating strategy for mature DCs. Fig. S12. Representative flow cytometry gating strategy for M1-like macrophages. Fig. S13. Confocal microscopic images of ROS-staining results of the tumor tissue slices after various treatments. Fig. S14. Representative flow cytometric plots and corresponding quantification results of DCs in the tumor tissues retrieved from mice after various treatments. Fig. S15. Representative flow cytometric plots and corresponding quantification results of M1-like macrophages in the tumor tissues retrieved from mice after various treatments. Fig. S16. Confocal fluorescence images of the immunofluorescence staining results of CD206 of the tumor tissue slices retrieved from mice after various treatments. Fig. S17. Confocal fluorescence images of the immunofluorescence staining results of CD4 and CD8 of the tumor tissue slices retrieved from mice in different groups. Fig. S18. Quantification of the flow cytometric results of MDSCs in the tumor tissues retrieved from [file research.0052.f1.docx]

**Supplementary Materials**

**Metal–Phenolic Network-Facilitated “Foe-to-Friend” Conversion of Melittin for Cancer Immunotherapy with Boosted Abscopal Effect**

*Yuxin Guo, Xinping Zhang, Shao-Zhe Wang, Hui-Heng Feng, Shun-Yu Wu, and Fu-Gen Wu**

State Key Laboratory of Bioelectronics, School of Biological Science and Medical Engineering, Southeast University, 2 Sipailou Road, Nanjing 210096, P. R. China

**1. Experimental**

***Materials***

Human serum albumin (HSA), lipopolysaccharide (LPS), fluorescein isothiocyanate (FITC), and ferric chloride hexahydrate (FeCl_3_•6H_2_O) were ordered from Sigma-Aldrich (Shanghai, China). Tannic acid (TA), bovine serum albumin (BSA), methylene blue (MB), and glutaraldehyde (50%, mass fraction) were obtained from Aladdin (Shanghai, China). Melittin (Mel) was synthesized by GL Biochem Ltd. (Shanghai, China). Resiquimod (R848) was obtained from MedChemExpress LLC (Shanghai, China). Dimethyl sulfoxide (DMSO) was purchased from Sinopharm Chemical Reagent Co., Ltd. (Shanghai, China). Collagenase IV was obtained from BioFroxx (Guangzhou, China). Deoxyribonuclease I was ordered from Leagene Biotechnology Co., Ltd. (Beijing, China). Methylthiazolyldiphenyl-tetrazolium bromide (MTT) was bought from Shanghai Yuanye Bio-Technology Co., Ltd. Interleukin-4 (IL-4) and granulocyte macrophage colony-stimulating factor (GM-CSF) were bought from Pepro Tech Inc. (Rocky Hill, USA). Anti-mouse calreticulin (CRT) (cat. no. 27298-1-ap) was purchased from Proteintech (Wuhan, China). Anti-mouse CD206 (cat. no. GB13438), and cyanine3 (Cy3)-labeled goat anti-rabbit IgG antibody were obtained from Wuhan Servicebio Technology Co., Ltd. (China). Anti-mouse CD3-PE (cat. no. 12-0032-82), anti-mouse CD4-FITC (cat. no. 11-0041-82), anti-mouse CD8a (CD8)-PE-cyanine7 (Cy7) (cat. no. 25-0081-82), anti-mouse forkhead box P3 (Foxp3)-PE-Cy7 (cat. no. 25-5773-82), anti-mouse Ly-6G/Ly-6C (Gr1)-FITC (cat. no. 11-5931-82), anti-mouse CD11b-PE (cat. no. 12-0112-82), anti-mouse CD11c-FITC (cat. no. 11-0114-82), anti-mouse CD80-PE (cat. no. 12-0801-82), anti-mouse F4/80-FITC (cat. no. 11-4081-82), and anti-mouse CD86-PE-Cy7 (cat. no. 25-0862-82) were bought from Invitrogen (Carlsbad, USA). Anti-mouse CD11c-PE (cat. no. 117308) was purchased from BioLegend (San Diego, USA). Mouse cytokine enzyme-linked immunosorbent assay (ELISA) kits for IL-6 (cat. no. 88-7064-22), IL-1β (cat. no. 88-7013-22), and tumor necrosis factor (TNF)-α (cat. no. 88-7324-22) were purchased from Invitrogen (Carlsbad, USA), and IL-10 (cat. no. EK210/4-96) and IFN-γ (cat. no. EK280/3-96) were bought from MultiSciences (Lianke) Biotech Co., Ltd. (Hangzhou, China). FITC-labeled goat anti-rabbit IgG antibody was obtained from EMD Millipore Corporation (Temecula, USA). Deionized water (18.2 MΩ•cm) was obtained from a Milli-Q system (Millipore, Billerica, MA).

***Investigation on the Formation mechanism of HRMTF NPs***

The formation mechanism of HRMTF NPs was investigated by separately mixing ethylenediaminetetraacetic acid disodium salt (EDTA, 200 mM), Triton X-100 (200 mM), urea (200 mM), or NaCl (200 mM) solution with the HRMTF suspensions (Mel: 40 μg/mL) in equal volumes, followed by measuring the hydrodynamic diameters of the resultant suspensions via a zetasizer instrument.

***Hemolysis assay***

The blood sample (2 mL) was collected from healthy BALB/c mice. Red blood cells (RBCs) were collected by centrifugation at 3000 rpm for 10 min, washed with phosphate-buffered saline (PBS) for 3 times, and resuspended using PBS (100 mL) to prepare the 2% RBC suspension. Then, different concentrations of HSA–R848–Mel–TA–Fe^3+^ (HRMTF) suspensions (Mel: 0, 2, 4, 8, 16, and 32 μg/mL; PBS as the solvent; 500 μL) were separately mixed with 2% RBC suspensions (500 μL) in centrifuge tubes. After being incubated at 37 ^o^C for 2 h, the supernatants were obtained through centrifugation at 3000 rpm for 5 min. RBCs in PBS and water were used as a negative control and a positive control, respectively.

***Cell culture and animal model***

4T1 (a murine mammary tumor cell line) and RAW 264.7 (mouse macrophage) cells were cultured in Roswell Park Memorial Institute (RPMI) 1640 (Gibco, USA) containing 10% fetal bovine serum (FBS), 0.08 mg/mL streptomycin, and 80 U/mL penicillin at 37 ^o^C in 5% CO_2_.

BALB/c mice (female, 6−8 weeks) were ordered from Yangzhou University Medical Center (Yangzhou, China). All the animal experiments were approved by the Animal Ethics Committee of Southeast University and were conducted in compliance with the Regulations for the Administration of Affairs Concerning Experimental Animals of China. The 4T1 xenograft tumor model was built by inoculating 2 × 10^6^ 4T1 cells onto the left flank of each BALB/c mouse (primary tumor) and inoculating 4 × 10^5^ 4T1 cells onto the right flank of each BALB/c mouse (distant tumor). All the experimental procedures regarding animals were performed under the approval of the Animal Care & Welfare Committee of Southeast University (No.20220516006) and in accordance with the Regulations for the Administration of Affairs Concerning Experimental Animals of China.

***Cell viability assay***

4T1 cells were seeded in 96-well plates at a density of 5 × 10^3^ cells/well. 24 h later, the cell culture media were replaced by fresh culture media containing various concentrations of R848, Mel, HSA–TA–Fe^3+^ (HTF), HSA–R848–TA–Fe^3+^ (HRTF), HSA–Mel–TA–Fe^3+^ (HMTF), or HRMTF. After another 24 h, MTT assay was performed to determine the relative cell viabilities. The viability of untreated cells (control) was set as 100%.

***Cellular internalization analysis of HRMTF***

For confocal imaging experiments, 4T1 cells were seeded in Lab-Tek 8-well chamber slides (1 × 10^4^ cells/well) and incubated for 1 d. For flow cytometric measurements, 4T1 cells were seeded in 24-well chamber slides (2.5 × 10^4^ cells/well) and incubated for 1 d. Confocal images were taken by a confocal microscope (TCS SP8, Leica, Germany). Flow cytometric analyses were performed using a flow cytometer (NovoCyte 2070R, ACEA Biosciences Inc., USA). FITC-labeled HSA (HSA-FITC) was used to prepare the FITC-labeled HRMTF (termed HRMTF-FITC).

To study the cellular internalization, the 4T1 cells were treated with HSA-FITC or HRMTF-FITC (Mel concentration: 4 µg/mL; HSA concentration: 20 µg/mL) for various time periods. Then, the cells were washed with PBS and observed by the confocal microscope at the excitation wavelengths of 488 nm or analyzed by the flow cytometer (FITC channel).

***Measurement of HO• generation***

The HTF suspension (100 μL, Fe^3+^: 40 μg/mL), H_2_O_2_ solution (10 μL, 800 mM), the MB solution (100 μL, 100 μg/mL), and 790 μL H_2_O were mixed, and the mixture was allowed to stand at room temperature for 2 h. The HO•-induced MB degradation was monitored by the change in the absorbance value at 665 nm via UV–vis spectroscopy. For comparison purpose, the HTF + MB suspension with the same MB and Fe^3+^ concentrations as those of the above HTF + H_2_O_2_ + MB suspension was also tested in a similar way.

***Intracellular reactive oxygen species (ROS) detection***

The production of ROS was determined by the ROS probe 2’,7’-dichlorodihydrofluorescein diacetate (DCFH-DA) (KeyGEN BioTECH, Nanjing, China). Specifically, 4T1 cells after different treatments were collected and treated with DCFH-DA (10 μM) for 30 min (37 ^o^C, 5% CO_2_). Then all the samples were measured by the flow cytometer and the confocal microscope.

***Apoptosis/necrosis assay***

To study the cellular apoptosis/necrosis induced by culture medium (control), R848, Mel, HTF, HMTF, HRTF, or HRMTF (with the same Mel concentration of 4 µg/mL or the R848 concentration of 1 µM), we carried out the annexin V-FITC/propidium iodide (PI) apoptosis detection assay. In brief, the 4T1 cells after various treatments were collected, washed 3 times with PBS, treated with the apoptosis detection kit (KeyGEN BioTECH, Nanjing, China), and then measured by the flow cytometer.

***Detection of immunogenic cell death (ICD) biomarkers***

The CRT expression on the cell membrane was studied via immunofluorescence and ﬂow cytometry. For immunofluorescence assay, 4T1 cells were seeded into Lab-Tek 8-well chamber slides at a density of 1 × 10^4^ cells per well and cultured for 1 d. After that, culture medium (control), R848, Mel, HTF, HMTF, HRTF, or HRMTF (with the same Mel concentration of 4 µg/mL or the R848 concentration of 1 µM) was added to the cells and incubated for 4 h. After being washed with PBS, the cells were fixed in 4% glutaraldehyde solution for 10 min. Next, the cells were blocked by 5% BSA for 3 h (25 ^o^C), treated with the anti-mouse CRT antibody (1/400) overnight (4 ^o^C), washed with PBS, and then treated with the FITC-labeled goat anti-rabbit IgG antibody (1/300) for another 3 h (25 ^o^C). After being washed with PBS and further stained with Hoechst 33342 (5 μg/mL) for 10 min, the cells were observed under the confocal microscope. The sample treatment procedures of the ﬂow cytometry analysis were similar to those of confocal microscopic imaging (except the Hoechst 33324 staining process).

The contents of the high-mobility group box 1 (HMGB1) and adenosine triphosphate (ATP) released by the 4T1 cells after different treatments were analyzed by the HMGB1 ELISA Kit (CUSABIO, Wuhan, China) and the Enhanced ATP Assay Kit (Beyotime, Shanghai, China), respectively, following the manufacturers’ protocols.

***Analyses of dendritic cell (DC) maturation and macrophage polarization in vitro***

Bone marrow-derived dendritic cells (BMDCs) were collected from femurs and tibias of BALB/c mice (female, 6−8 weeks of age). Brieﬂy, the mouse bone marrow cells were collected and incubated in the X-VIVO 15 culture medium (Lonza, Switzerland) with IL-4 (10 ng/mL) and GM-CSF (20 ng/mL) for 5 days to obtain the immature BMDCs. The immature BMDCs were seeded in Lab-Tek 8-well chamber slides (1 × 10^4^ cells/well) and incubated for 24 h. After that, the culture media were replaced by fresh culture media containing various drugs (e.g., culture medium (control), R848, Mel, HTF, HMTF, HRTF, or HRMTF) (with the same Mel concentration of 2 µg/mL or the R848 concentration of 0.5 µM) and incubated for another 24 h. Next, BMDCs were collected and stained with anti-mouse CD11c-FITC, anti-mouse CD86-PE-Cy7, and anti-mouse CD80-PE, and analyzed by ﬂow cytometry. The immature BMDCs treated with LPS (25 ng/mL) and GM-CSF (20 ng/mL) were set as positive control and negative control, respectively.

For the macrophage polarization experiment, the procedures were similar to those in the above experiment, and M2-like RAW 264.7 cells (pretreated with IL-4) were used to replace the above immature BMDCs. The RAW 264.7 cells treated with different drugs (e.g., culture medium (control), R848, Mel, HTF, HMTF, HRTF, or HRMTF) (with the same Mel concentration of 2 µg/mL or the R848 concentration of 0.5 µM) were stained with anti-mouse F4/80-FITC and anti-mouse CD11c-PE antibodies, and then analyzed by ﬂow cytometry. The RAW 264.7 cells treated with culture medium, IL-4 (20 ng/mL), and “IFN-γ (100 ng/mL) + LPS (25 ng/mL)” were set as the M0, M2, and M1 types of macrophages, respectively.

To evaluate the influence of the “secretome” of 4T1 cells after different treatments on BMDCs and RAW 264.7 cells, 4T1 cells were seeded in Lab-Tek 8-well chamber slides (1 × 10^4^ cells/well) and incubated for 24 h. Then, the culture media were replaced by fresh culture media containing R848, Mel, HTF, HMTF, HRTF, or HRMTF (with the same Mel concentration of 4 µg/mL or the R848 concentration of 1 µM; culture medium was used as the control group) and incubated for 4 h. Then, the culture media were replaced by fresh culture media and incubated for 20 h. Next, the above culture media were used to incubate immature BMDCs or M2-like RAW 264.7 cells for 1 day. Finally, the treated BMDCs and RAW 264.7 cells were stained by antibodies (using the same antibodies described in the above two paragraphs) and analyzed by ﬂow cytometry.

***In vivo antitumor experiments***

The 4T1 tumor-bearing BALB/c mice were divided into 7 groups (*n* = 5/group) for different treatments as follows: (1) intratumorally (*i*.*t*.) injected with PBS, (2) *i*.*t*. injected with Mel solution, (3) *i*.*t*. injected with R848 solution, (4) *i*.*t*. injected with HTF suspension, (5) *i*.*t*. injected with HMTF suspension, (6) *i*.*t*. injected with HRTF suspension, and (7) *i*.*t*. injected with HRMTF suspension. Note that the groups 2–7 had the same Mel dose of 5 mg/kg or the R848 dose of 1.25 µmol/kg. The tumor size and body weight were recorded for 30 d. Tumor volume (*V*) was calculated as width^2^ × length/2.

***Tumor immunofluorescence analyses and histological staining of tumor tissues***

Briefly, the 4T1 tumor-bearing BALB/c mice were sacrificed after various treatments, and their tumors were excised for the immunofluorescence analyses of CRT, CD4, CD8, and CD206, the ROS analysis (via the dihydroethidium (DHE) assay), and the hematoxylin and eosin (H&E) assay following standard protocols.

For terminal deoxynucleotidyl transferase (TdT)-mediated dUTP nick end labeling (TUNEL) assay, a TUNEL apoptosis in situ detection kit (KeyGEN BioTECH, Nanjing, China) was used following the instructions from the manufacturer.

***Flow cytometry analyses of tumor tissues and lymph nodes (LNs)***

The tumor tissues and LNs of 4T1 tumor-bearing BALB/c mice after different treatments were retrieved for flow cytometry analysis. The tumor tissues were cultured in the dissociation buffer (100 μg/mL deoxyribonuclease I and 1 mg/mL collagenase IV) at 37 ^o^C for 30 min. The LNs were stored in PBS. The above treated tumor tissues and LNs were passed through cell strainers (70 μm) to obtain the single-cell suspensions, which were washed with 1 wt% BSA buffer (solvent: PBS), and then stained with anti-CD11c-FITC, anti-CD80-PE, anti-CD86-PE-Cy7, anti-CD11c-PE, anti-F4/80-FITC, anti-CD3-PE, anti-CD4-FITC, and anti-CD8-PE-Cy7 following the instructions of the manufacturers to detect the maturation of DCs (CD11c^+^CD86^+^CD80^+^), the M1-like macrophages (CD11c^+^F4/80^+^), the helper T cells (CD3^+^CD4^+^), and the cytotoxic T cells (CD3^+^CD8^+^), respectively. All the antibodies were used at a dilution of 1:200. The antibodies-stained single-cell suspensions were analyzed by flow cytometry.

***Cytokine profile analysis***

The ELISA analysis of intratumoral levels of IL-6, IFN-γ, IL-1β, TNF-α, and IL-10 as well as the plasma levels of TNF-α, IFN-γ, and IL-10 were performed following the instructions of the ELISA kits.

***Histological staining of major organs and blood hemanalysis***

The BALB/c mice were *i*.*t*. injected with PBS (control), Mel, or HRMTF. 14 days after administration, the major organs of the treated mice were collected for H&E assay following standard protocols. Besides, the routine blood analyses were carried out by an automatic hematology analyzer (BC-2800Vet, Mindray, China), and the biochemical tests were performed on an automated biochemical analyzer (SMT-100V, Seamaty, China).

***Statistical analysis***

Most of the numeric data were expressed as mean ± standard deviation (s.d.). The significance between two groups was analyzed by two-tailed Student’s *t*-test. For multiple comparisons, one-way analysis of variance (ANOVA) with Tukey’s post-hoc test was adopted. *P* values of less than 0.05 were considered significant. **P* < 0.05, ***P* < 0.01, ****P* < 0.001, *****P* < 0.0001.

1. **Supporting figures**

**
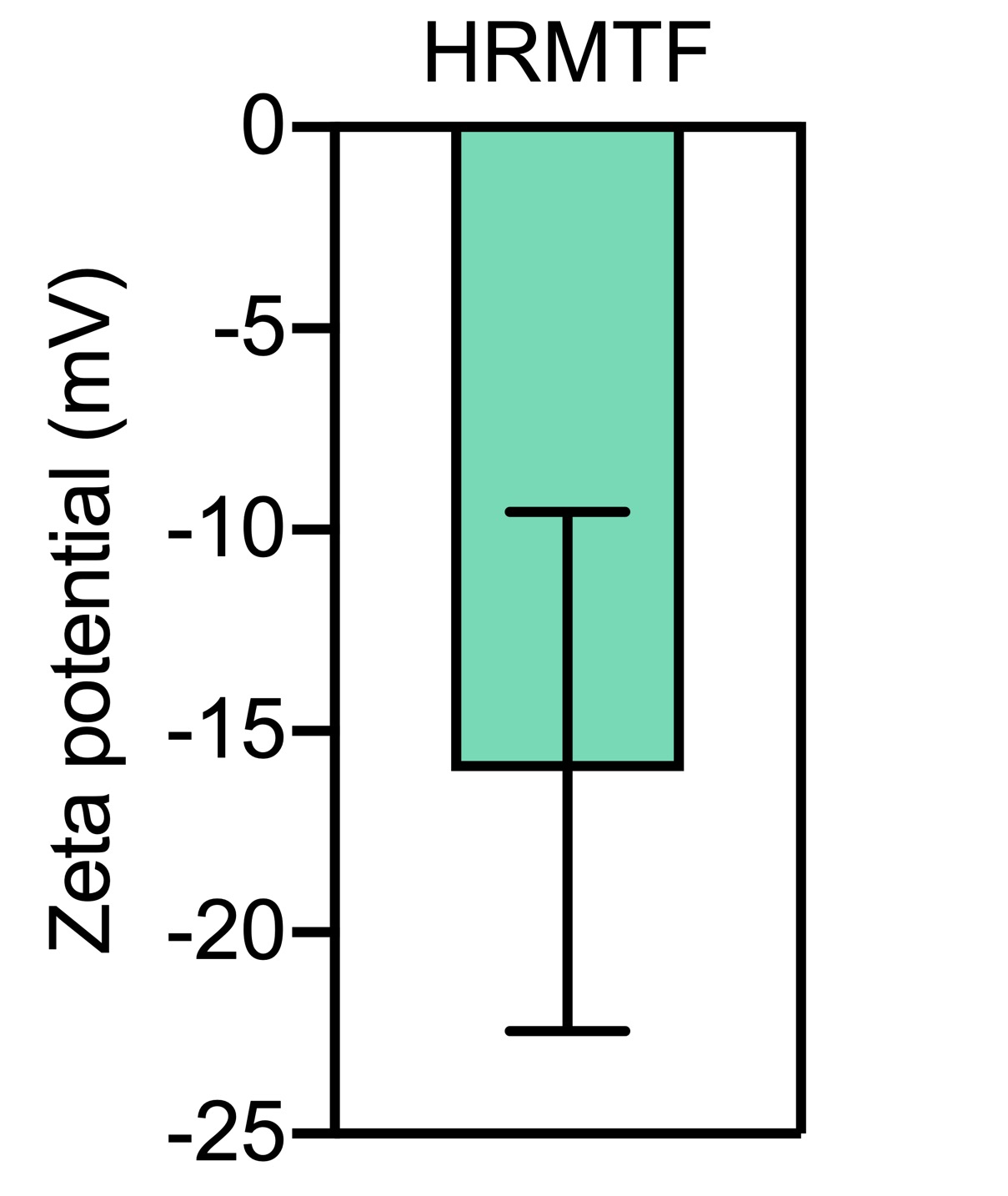
**

**Figure S1.** Zeta potential of HRMTF nanoparticles (NPs).


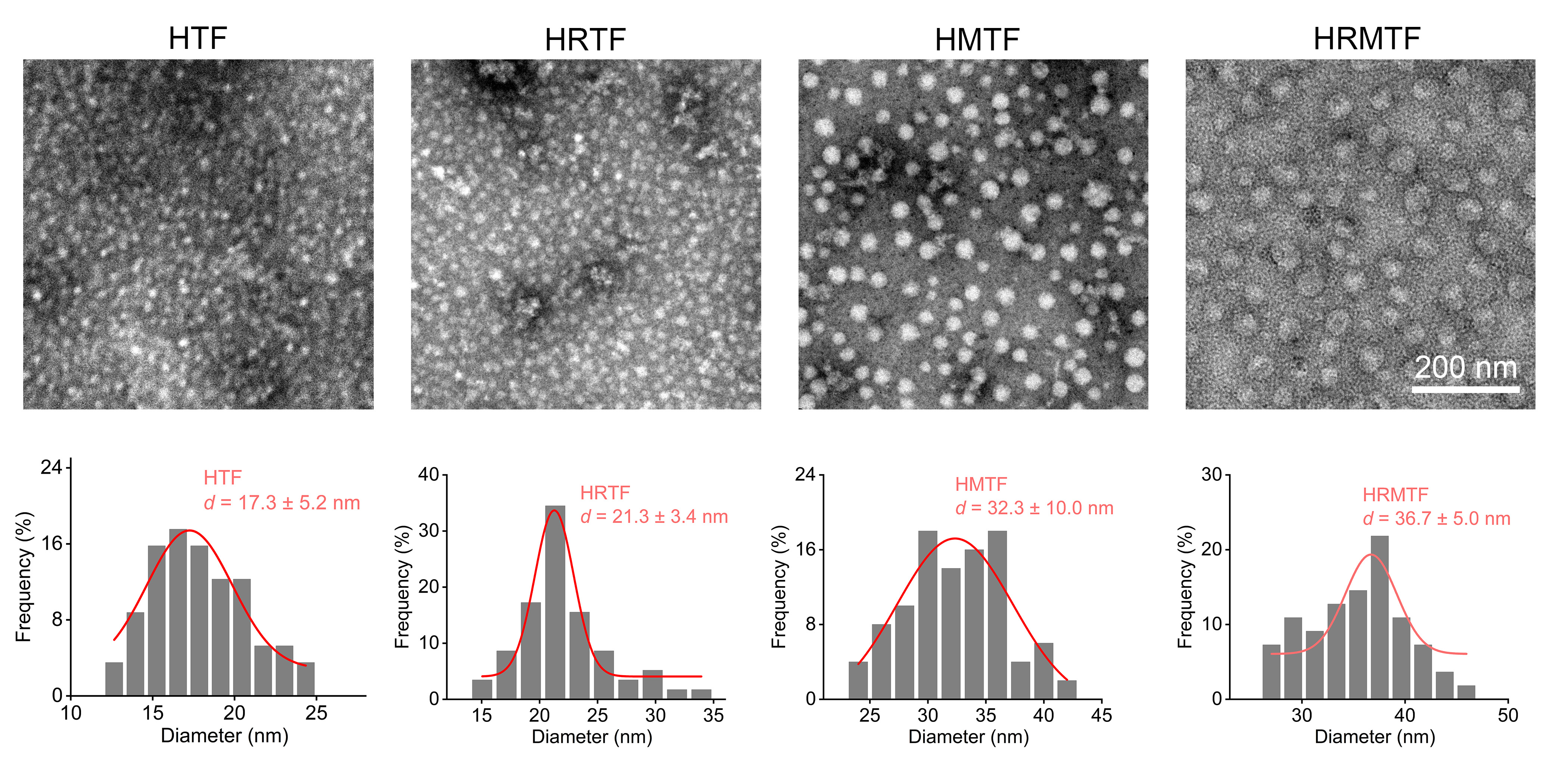


**Figure S2.** TEM images and corresponding size distribution results of HTF, HRTF, and HMTF NPs.


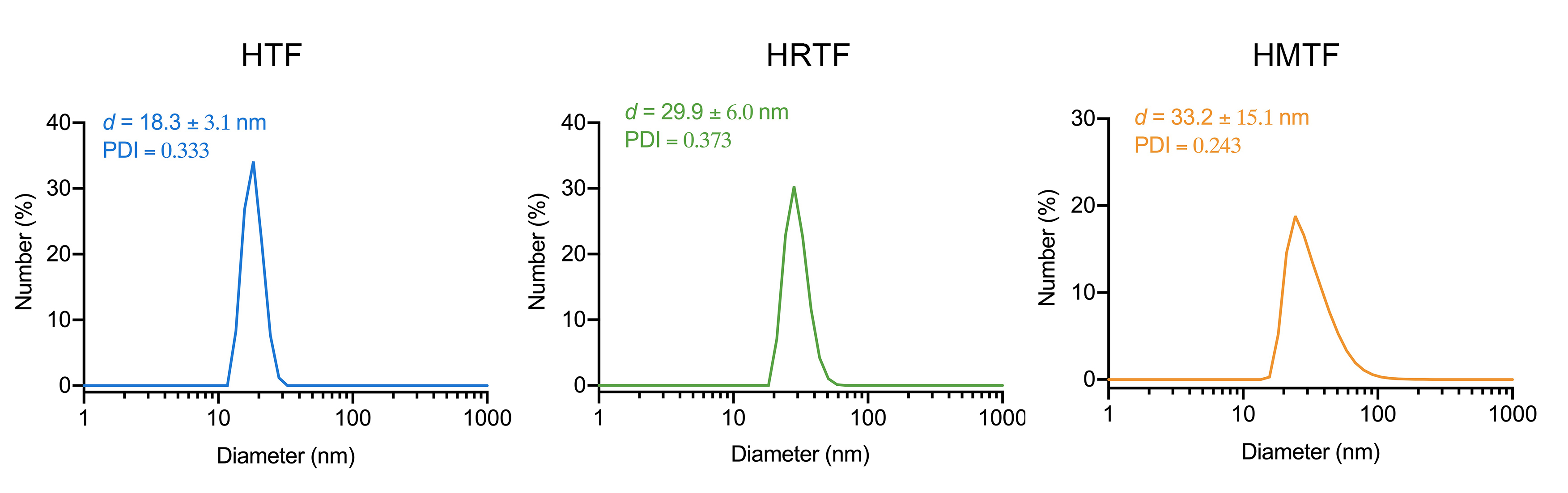


**Figure S3.** Hydrodynamic diameters of HTF, HRTF, and HMTF NPs.

**
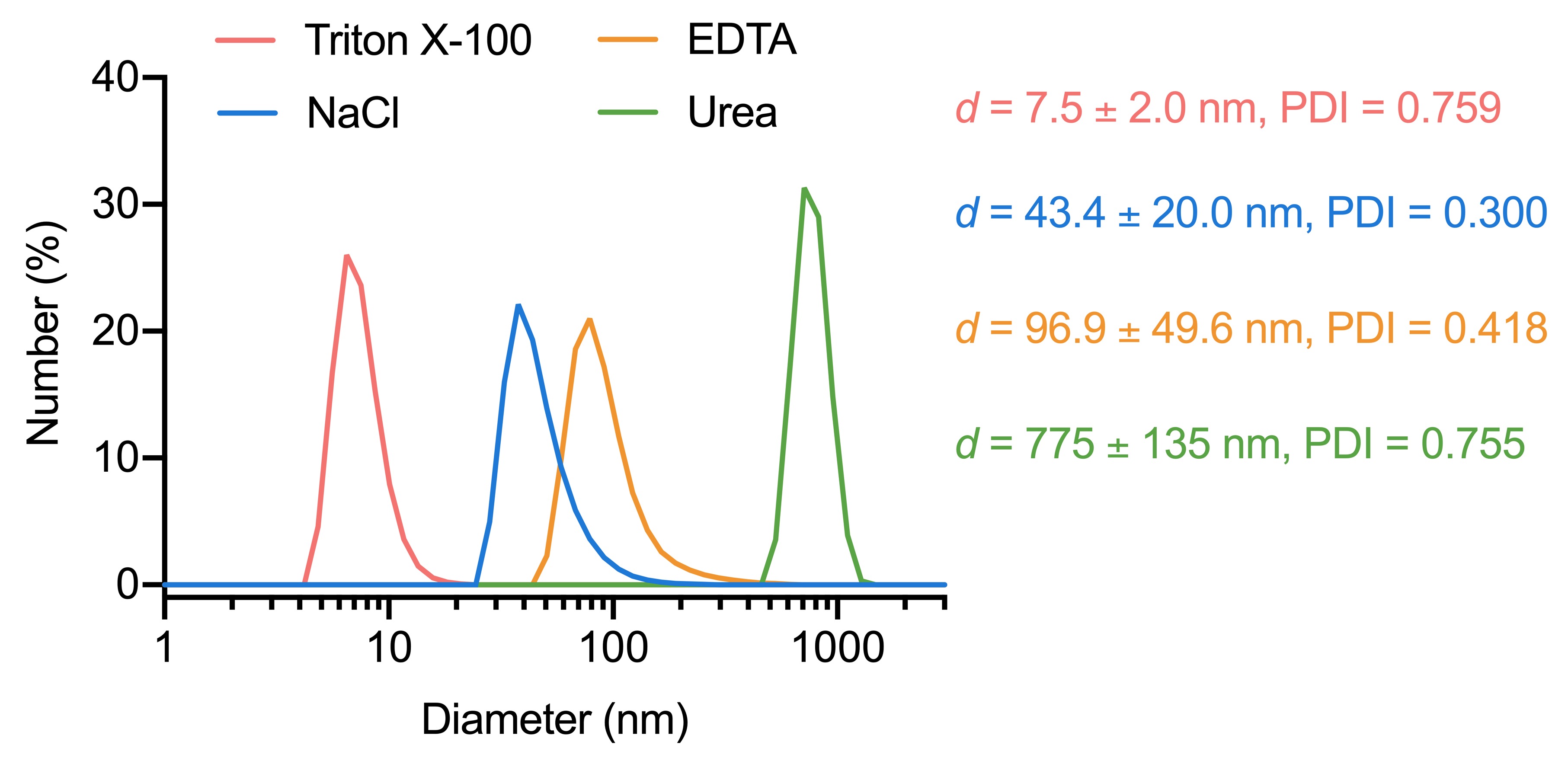
**

**Figure S4.** Hydrodynamic diameters of HRMTF NPs after the treatment of Triton X-100, NaCl, EDTA, or urea.

**
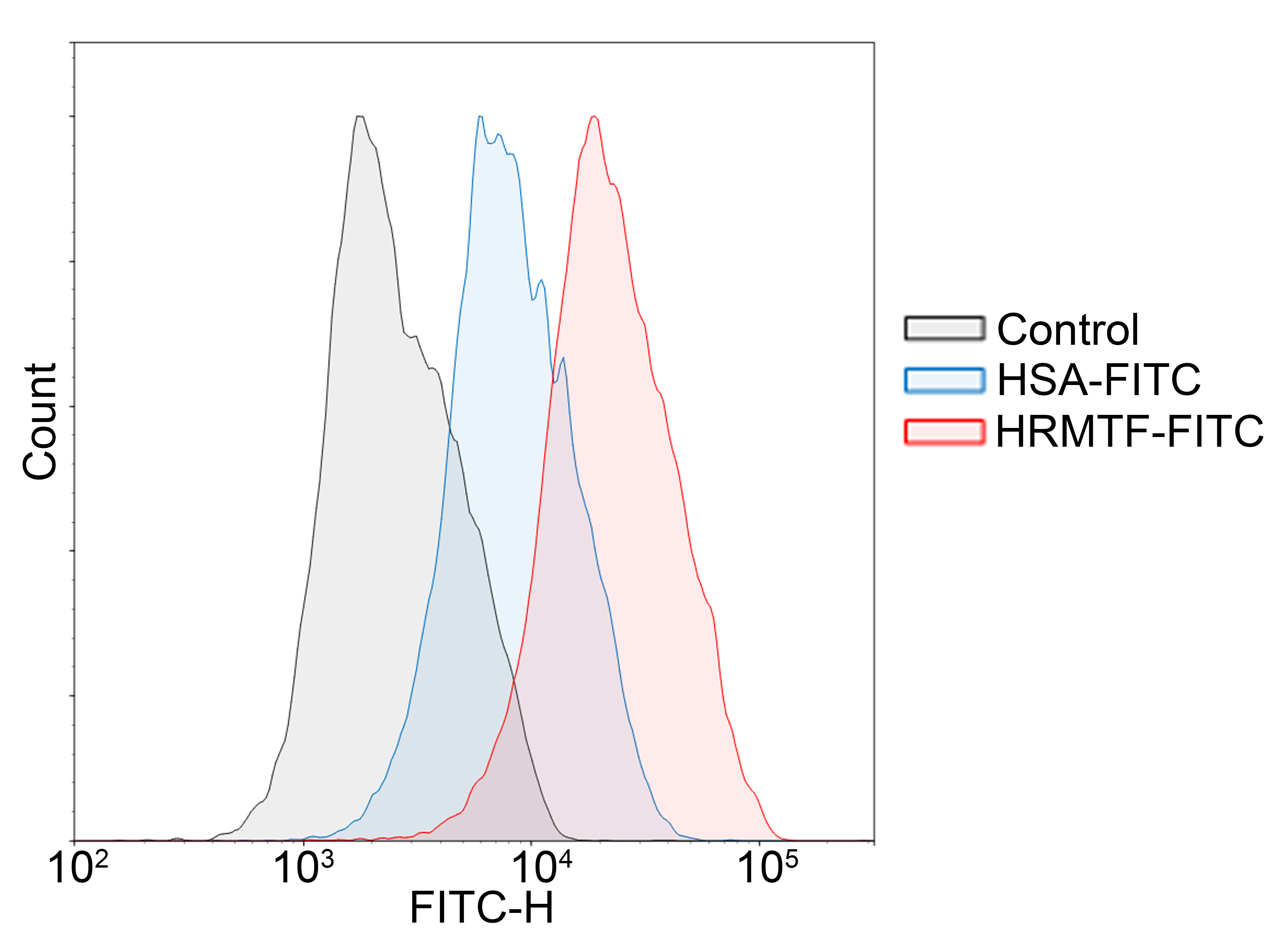
**

**Figure S5.** Representative flow cytometric results showing the FITC fluorescence intensities in the 4T1 cells after incubation with HSA-FITC or HRMTF-FITC for 2 h. The Mel concentration in HRMTF-FITC was 4 μg/mL.

**
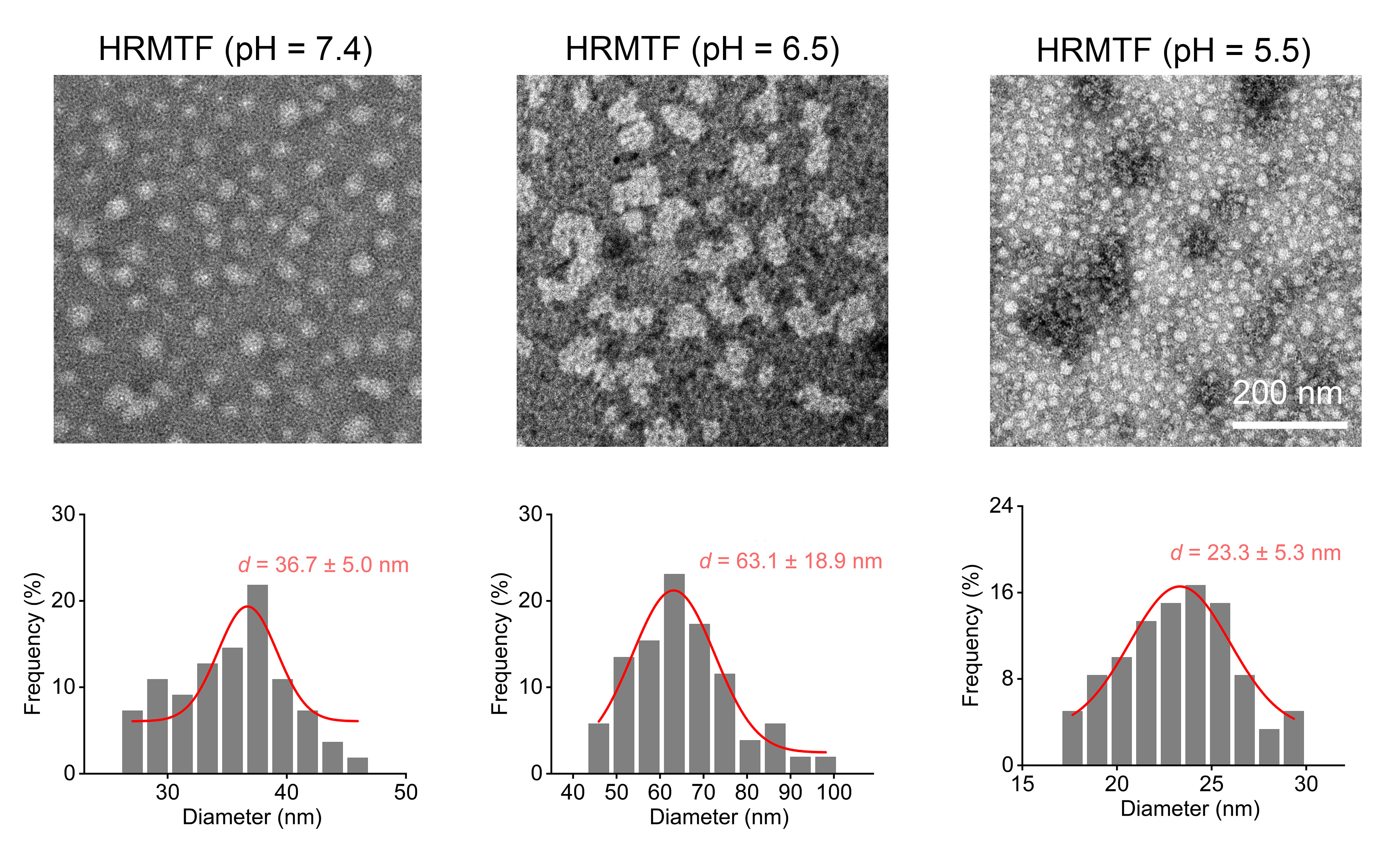
**

**Figure S6.** TEM images and corresponding size distribution results of HRMTF NPs at pH 7.4, 6.5, and 5.5, respectively. The pH 7.4 results were extracted from Figure 1a and 1b.


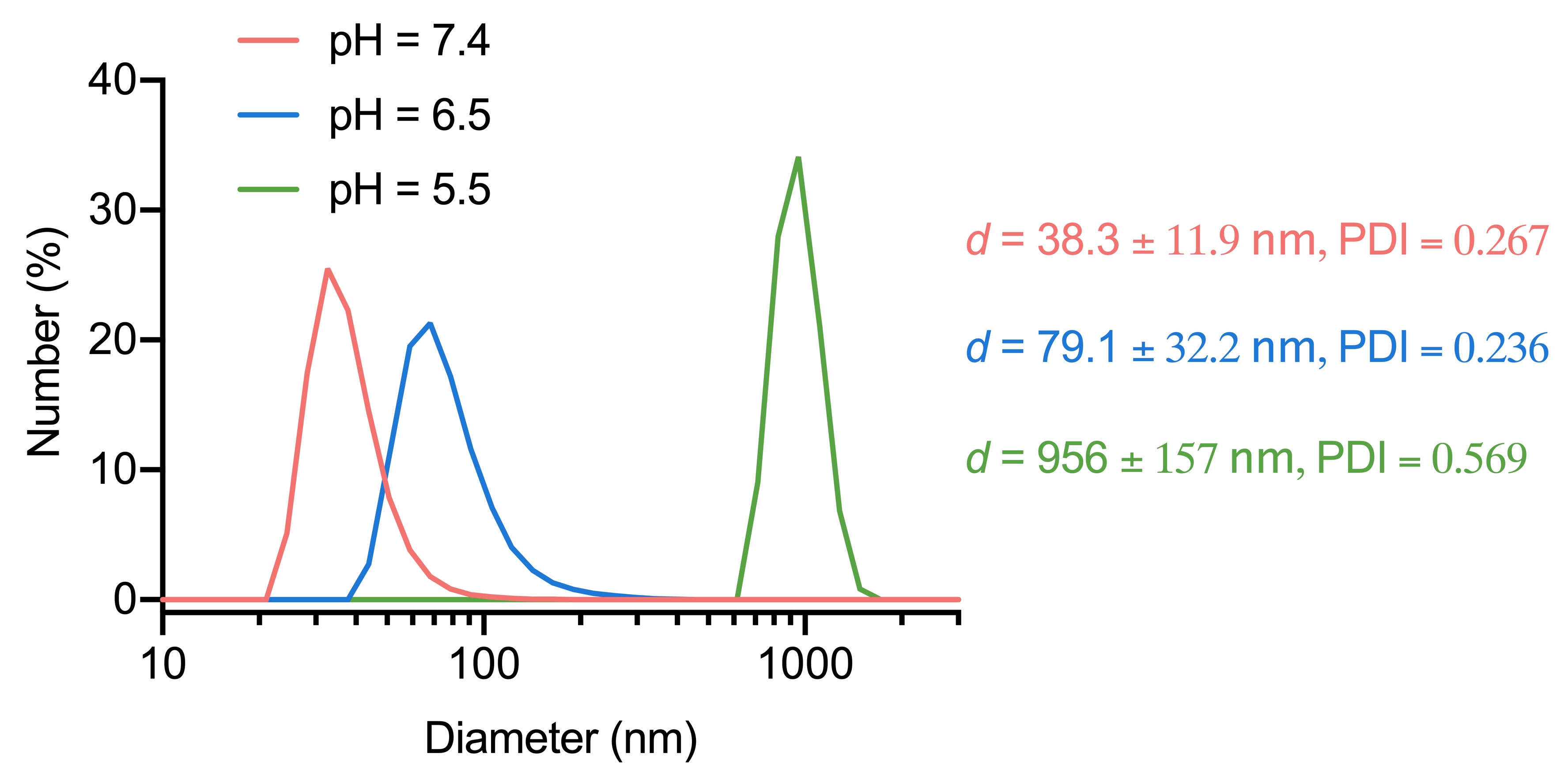


**Figure S7.** Hydrodynamic diameters of HRMTF NPs at pH 7.4, 6.5, and 5.5, respectively. The pH 7.4 result was extracted from Figure 1c.


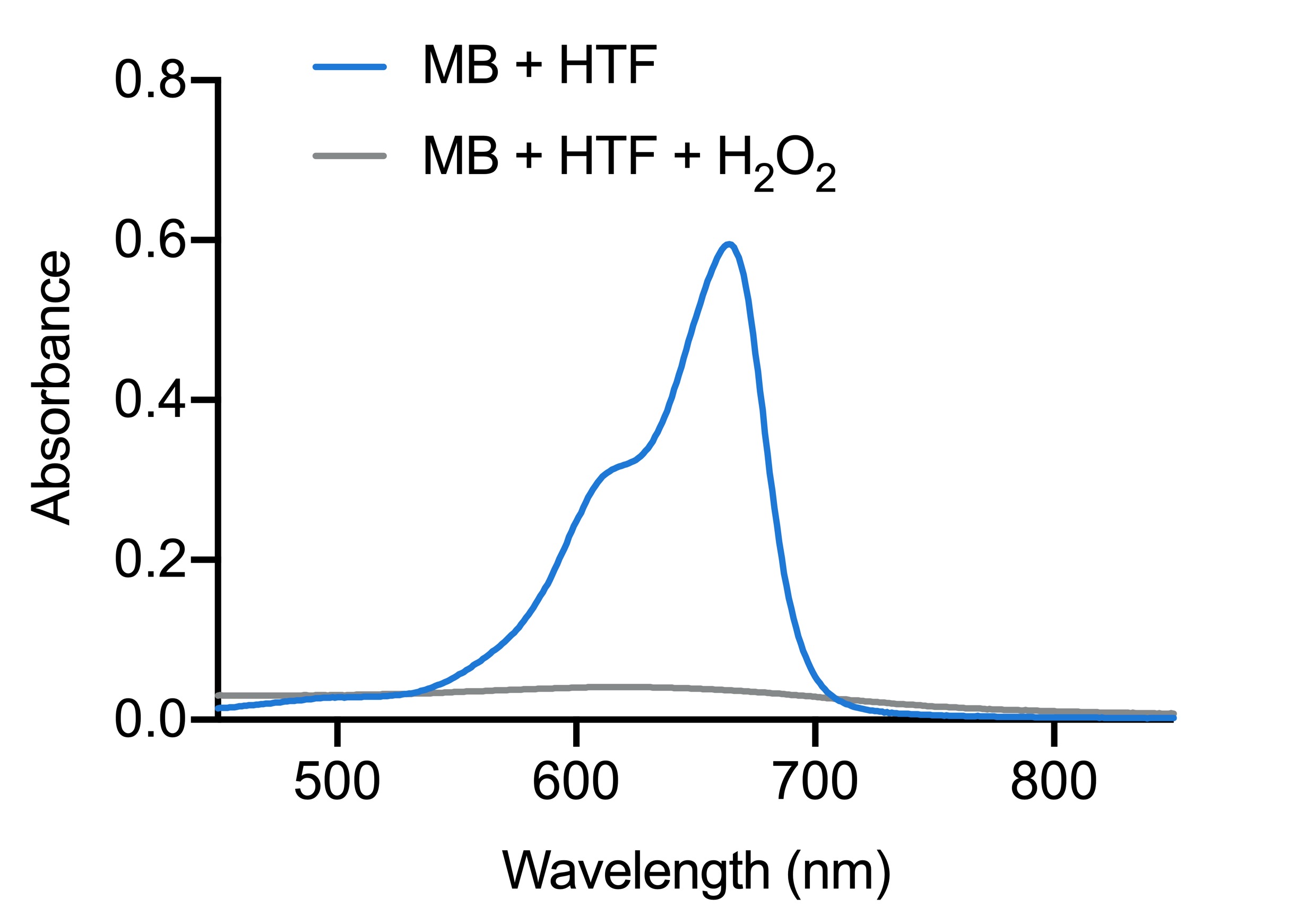


**Figure S8.** UV–vis absorption spectra of the solutions containing MB + HTF or MB + HTF + H_2_O_2_.


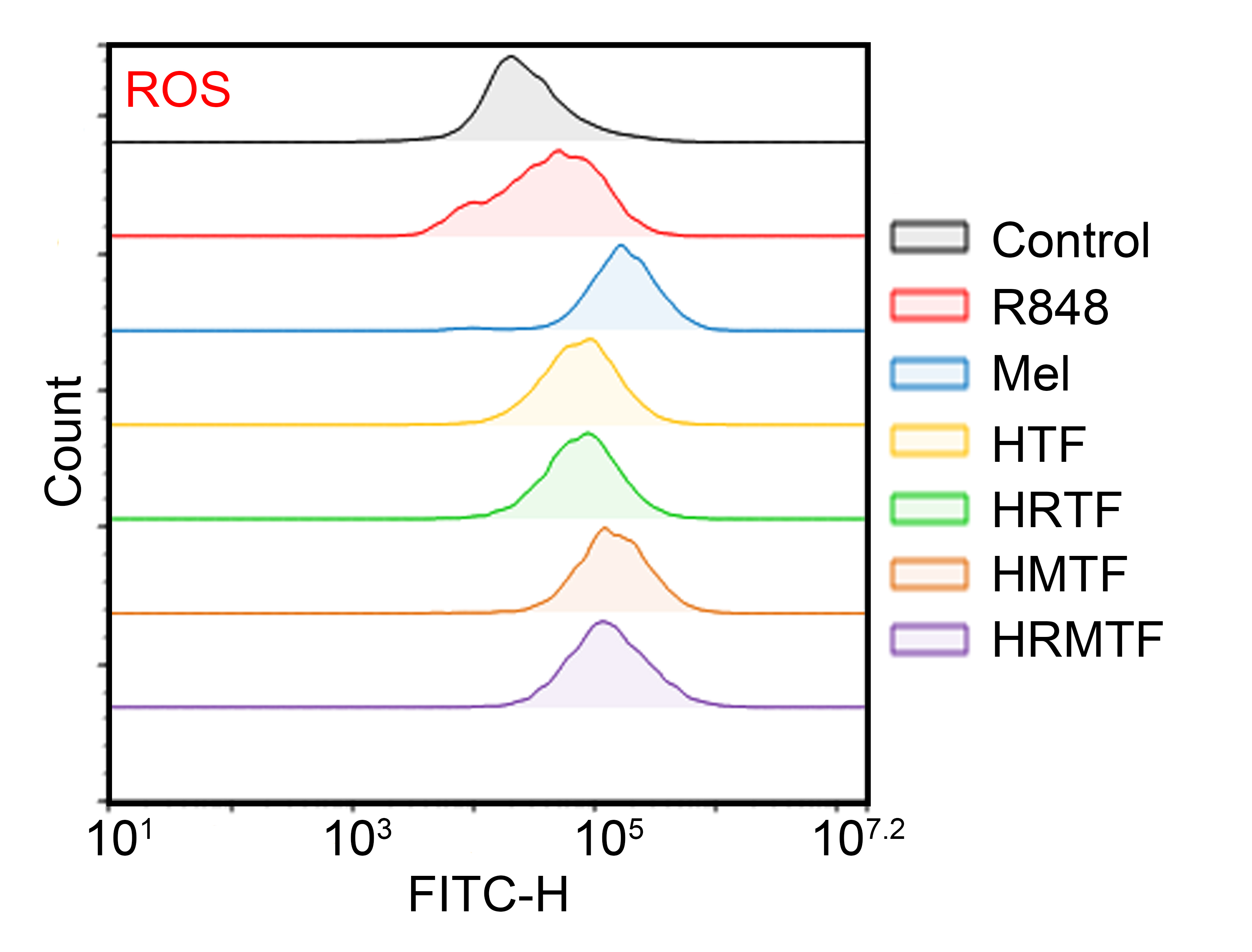


**Figure S9.** Representative flow cytometric results showing the ROS levels in the 4T1 cells after various treatments. The Mel content in all the samples was fixed at 4 μg/mL.


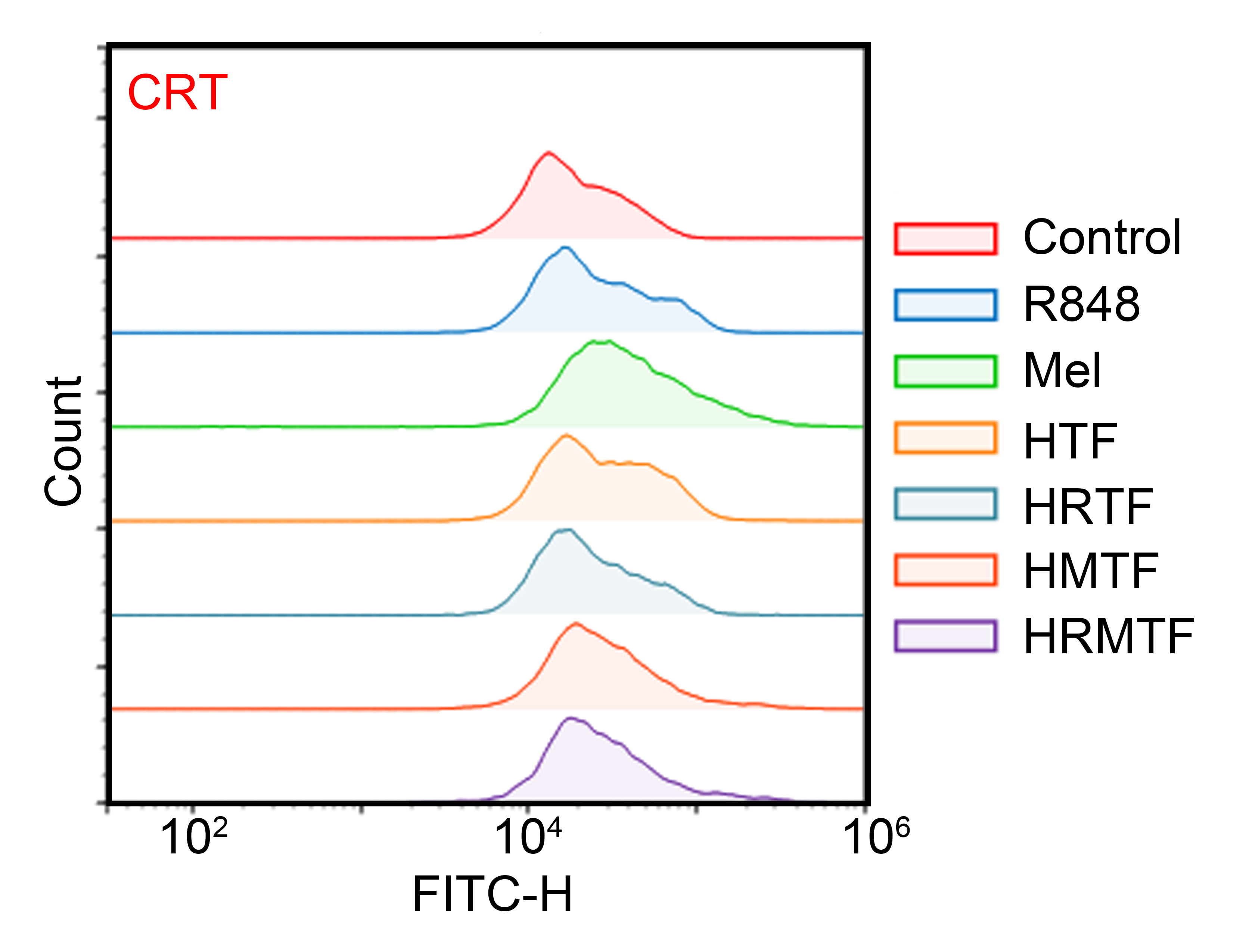


**Figure S10.** Representative flow cytometric results showing the CRT levels on the 4T1 cells after various treatments. The Mel content in all the samples was fixed at 4 μg/mL.


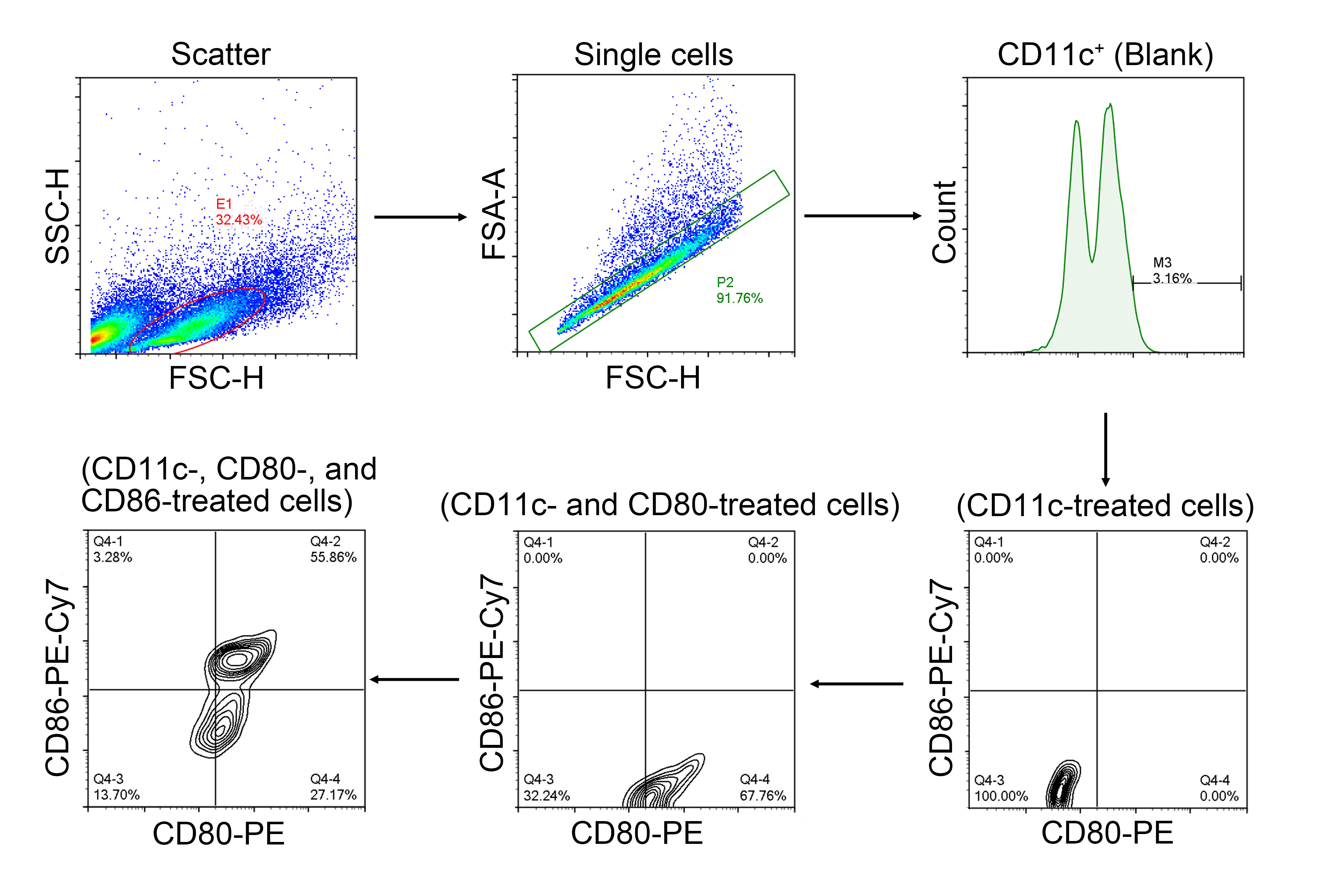


**Figure S11.** Representative flow cytometry gating strategy for CD11c^+^CD80^+^CD86^+^ mature DCs. The experiment was performed twice with similar results.


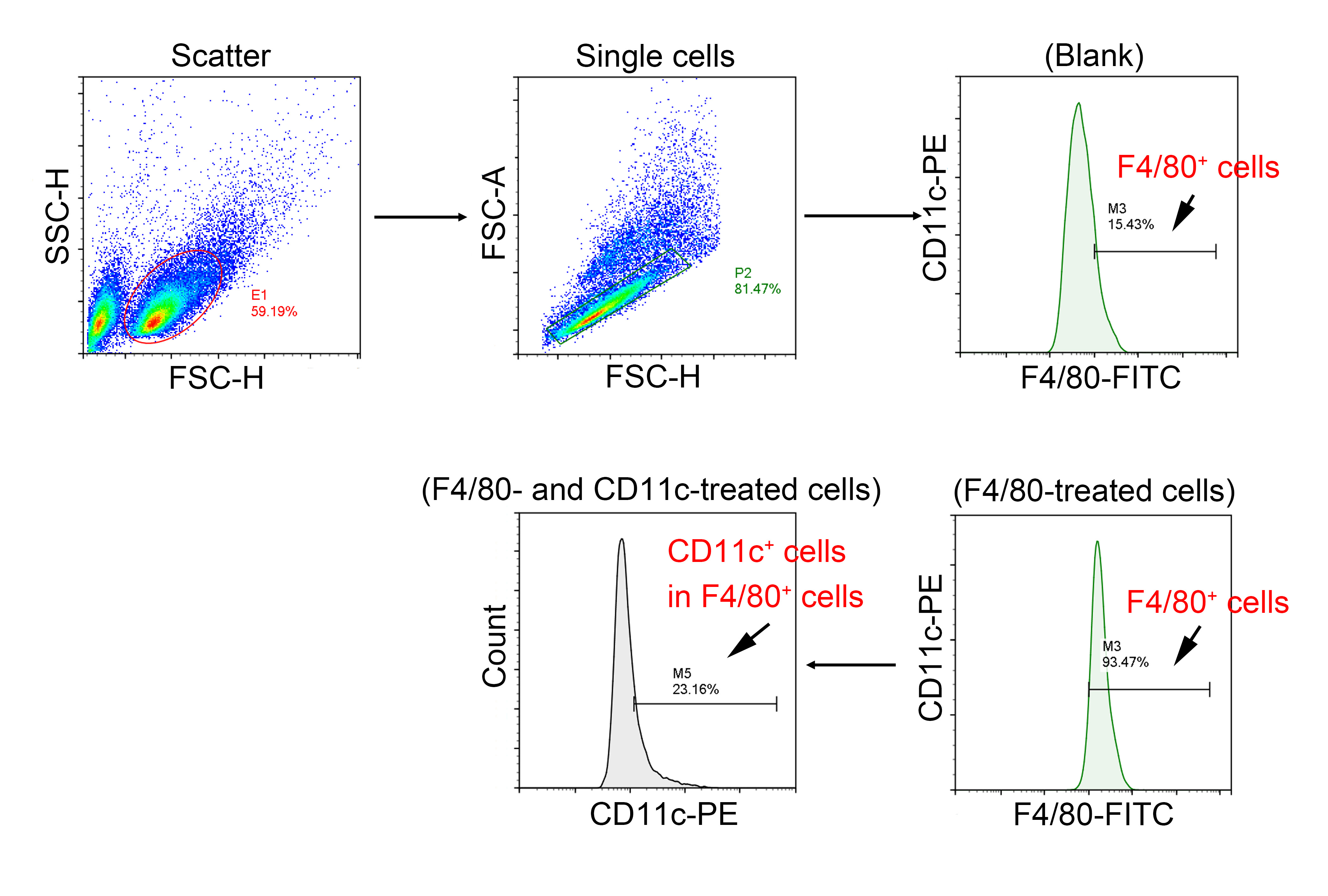


**Figure S12.** Representative flow cytometry gating strategy for CD11c^+^F4/80^+^ M1-like macrophages. The experiment was performed twice with similar results.


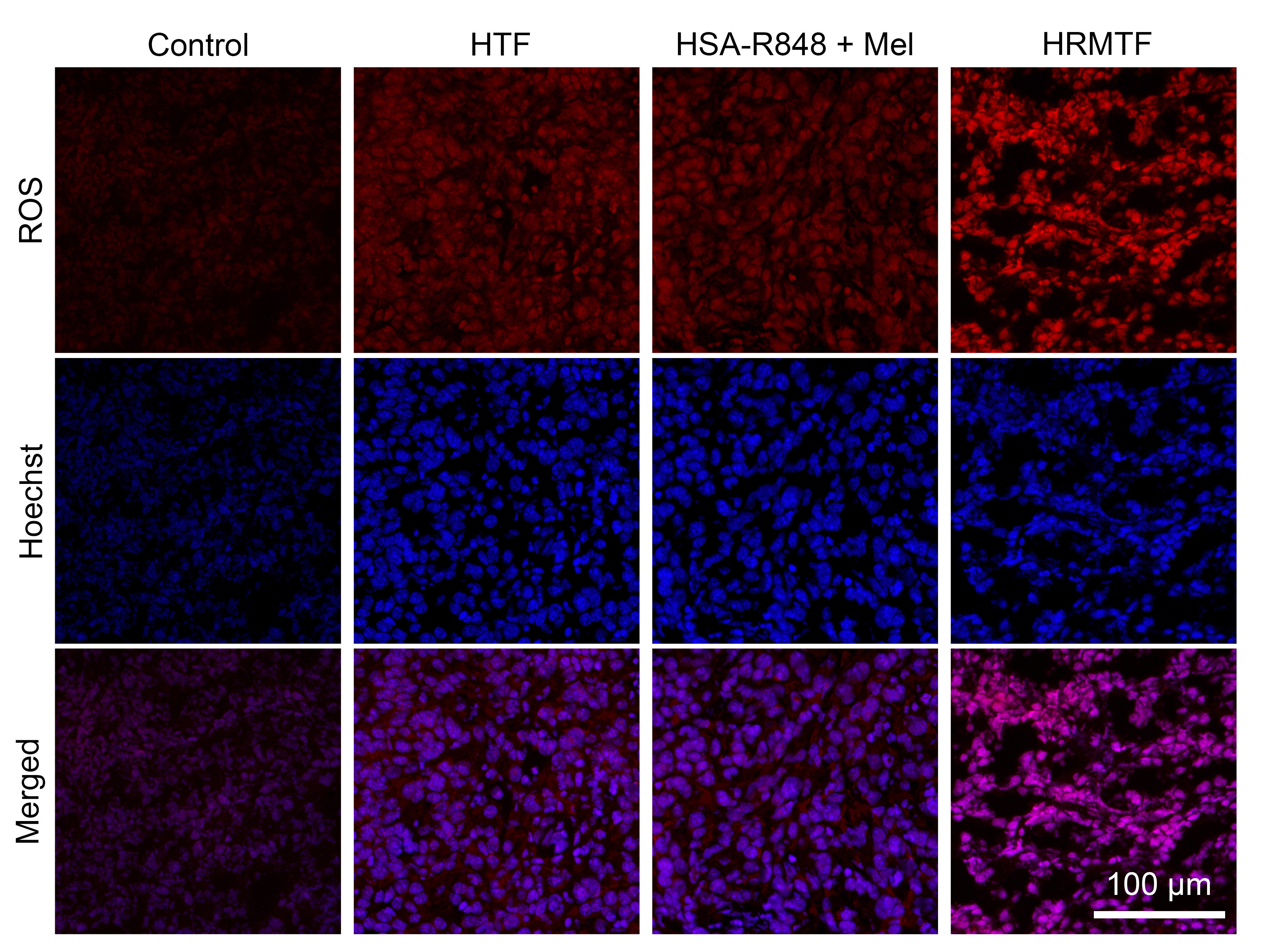


**Figure S13.** Confocal microscopic images of representative ROS staining results of the tumor tissue slices from the primary tumor of the 4T1 tumor-bearing BALB/c mice taken at day 6 after various treatments. Before imaging, the cell nuclei were stained with Hoechst 33342 (abbreviated as Hoechst). Scale bar: 100 μm.


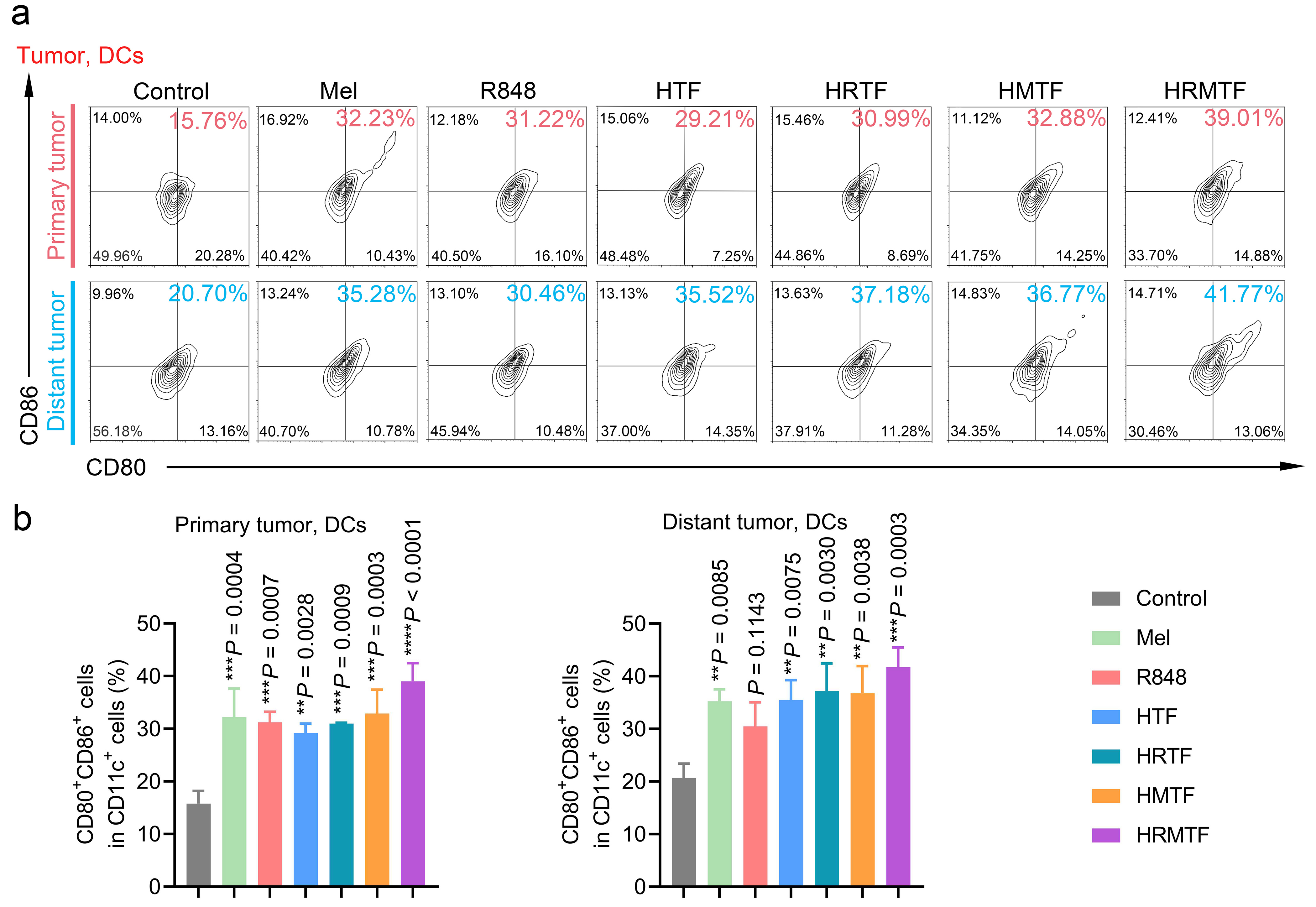


**Figure S14.** Representative flow cytometric plots (a) and corresponding quantification results (b) of DCs (CD11c^+^CD80^+^CD86^+^) in the tumor tissues retrieved from the bilateral 4T1 tumor-bearing BALB/c mice 7 d after different treatments. ***P* < 0.01, ****P* < 0.001, *****P* < 0.0001.


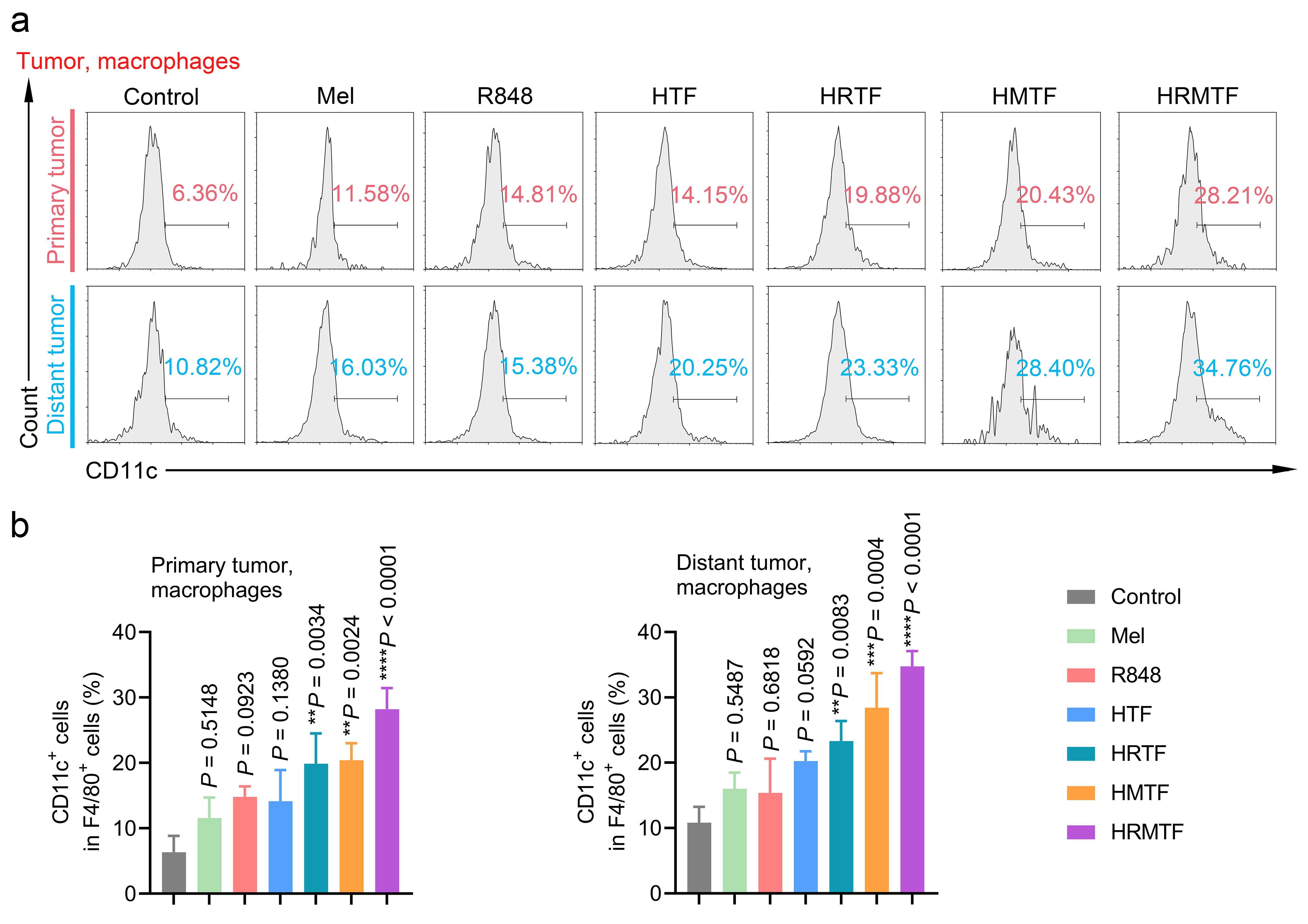


**Figure S15.** Representative flow cytometric plots (a) and corresponding quantification results (b) of M1-like macrophages (F4/80^+^CD11c^+^) in the tumor tissues retrieved from the bilateral 4T1 tumor-bearing BALB/c mice 7 d after different treatments. ***P* < 0.01, ****P* < 0.001, *****P* < 0.0001.


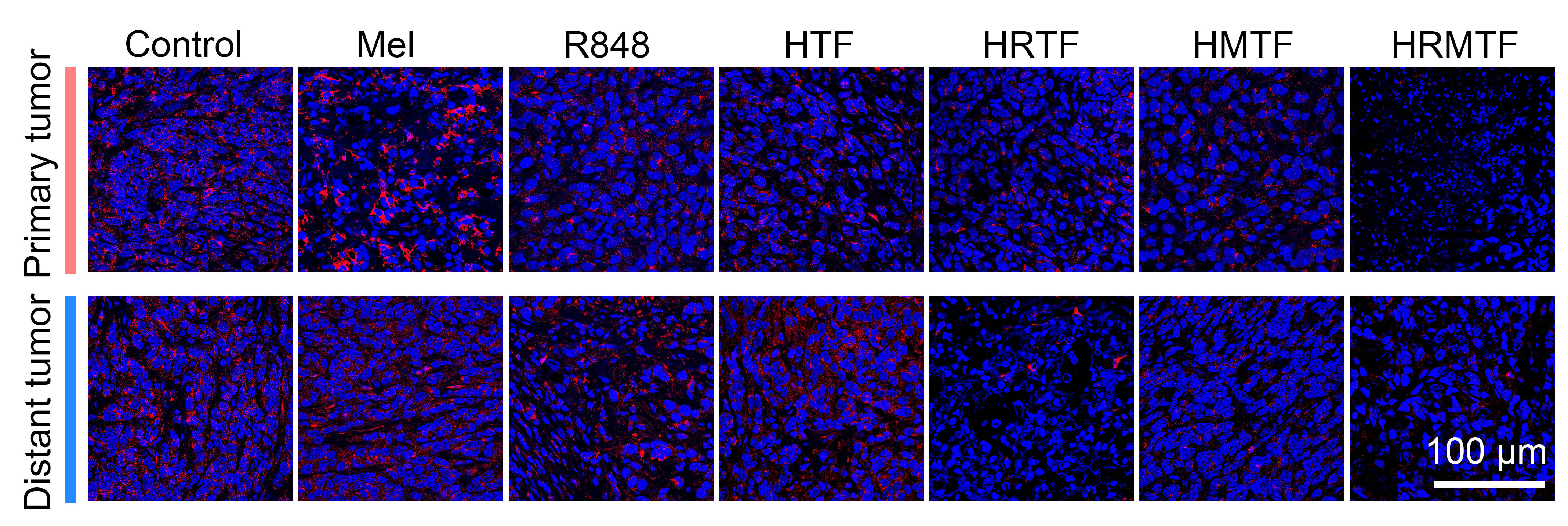


**Figure S16.** Confocal fluorescence images of the immunofluorescence staining results of CD206 of the tumor tissue slices from the bilateral 4T1 tumor-bearing BALB/c mice sacrificed at day 7 after different treatments. Scale bar: 100 μm.


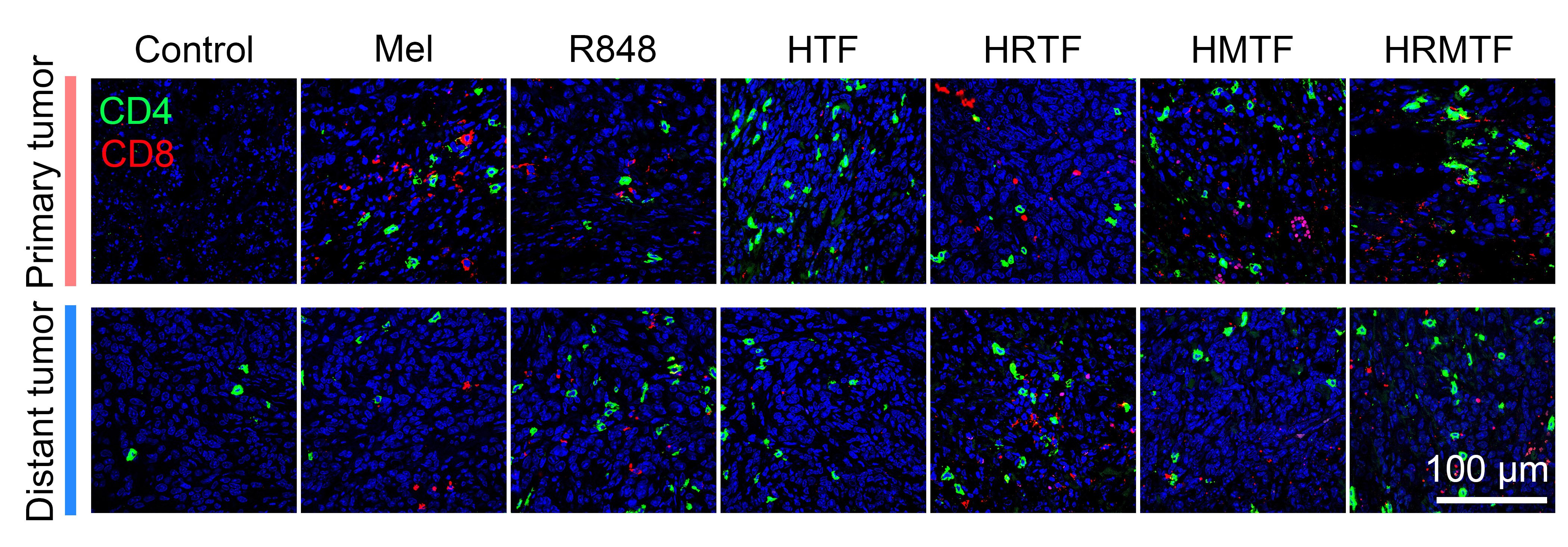


**Figure S17.** Confocal fluorescence images of the immunofluorescence staining results of CD4 (green) and CD8 (red) of the tumor tissue slices from the bilateral 4T1 tumor-bearing BALB/c mice sacrificed at day 7 after different treatments. Scale bar: 100 μm.


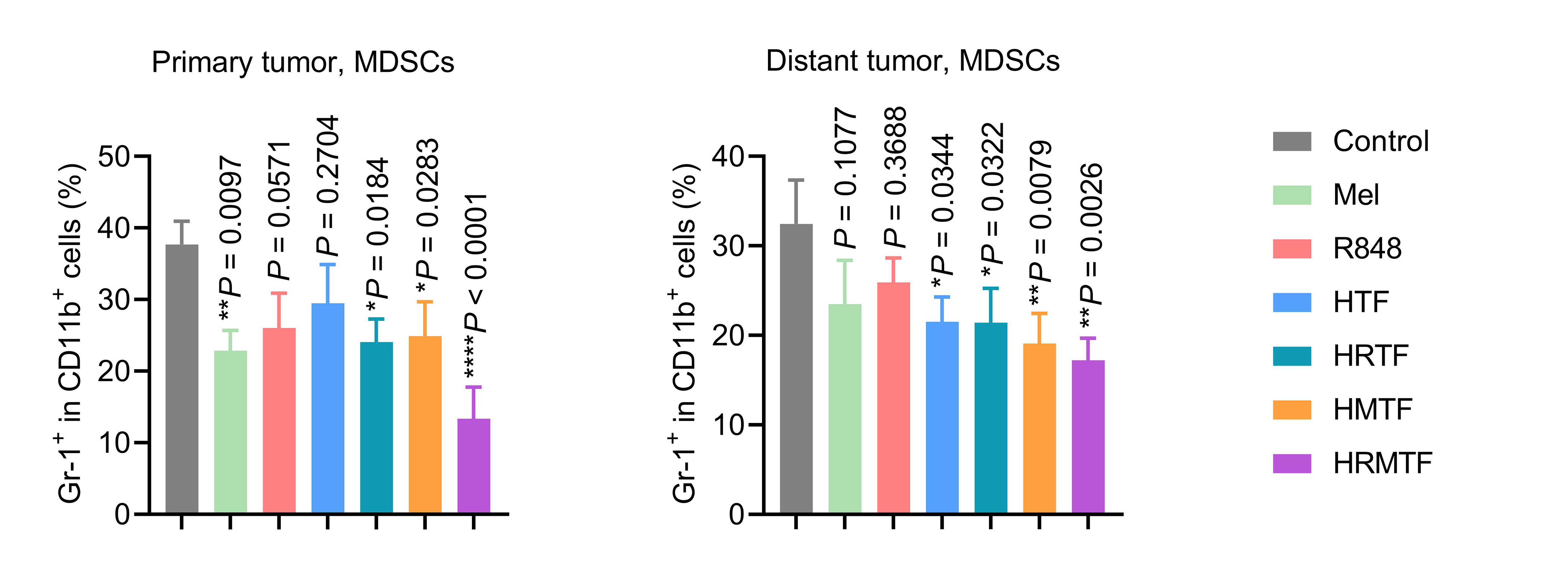


**Figure S18.** Quantification of the flow cytometric results of MDSCs (CD11b^+^Gr-1^+^) in the tumor tissues retrieved from the bilateral 4T1 tumor-bearing BALB/c mice 7 d after different treatments. **P* < 0.05, ***P* < 0.01, *****P* < 0.0001.


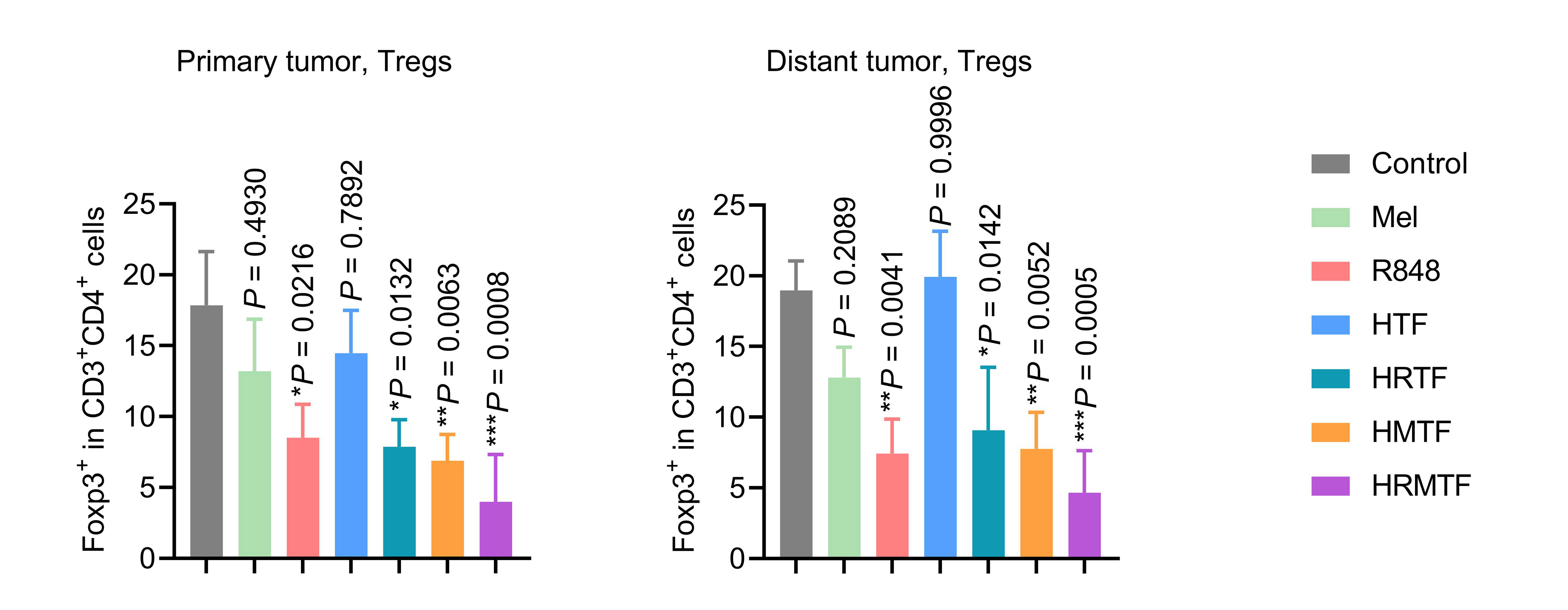


**Figure S19.** Quantification of the flow cytometric results of Tregs (CD3^+^CD4^+^Foxp3^+^) in the tumor tissues retrieved from the bilateral 4T1 tumor-bearing BALB/c mice 7 d after different treatments. **P* < 0.05, ***P* < 0.01, ****P* < 0.001.


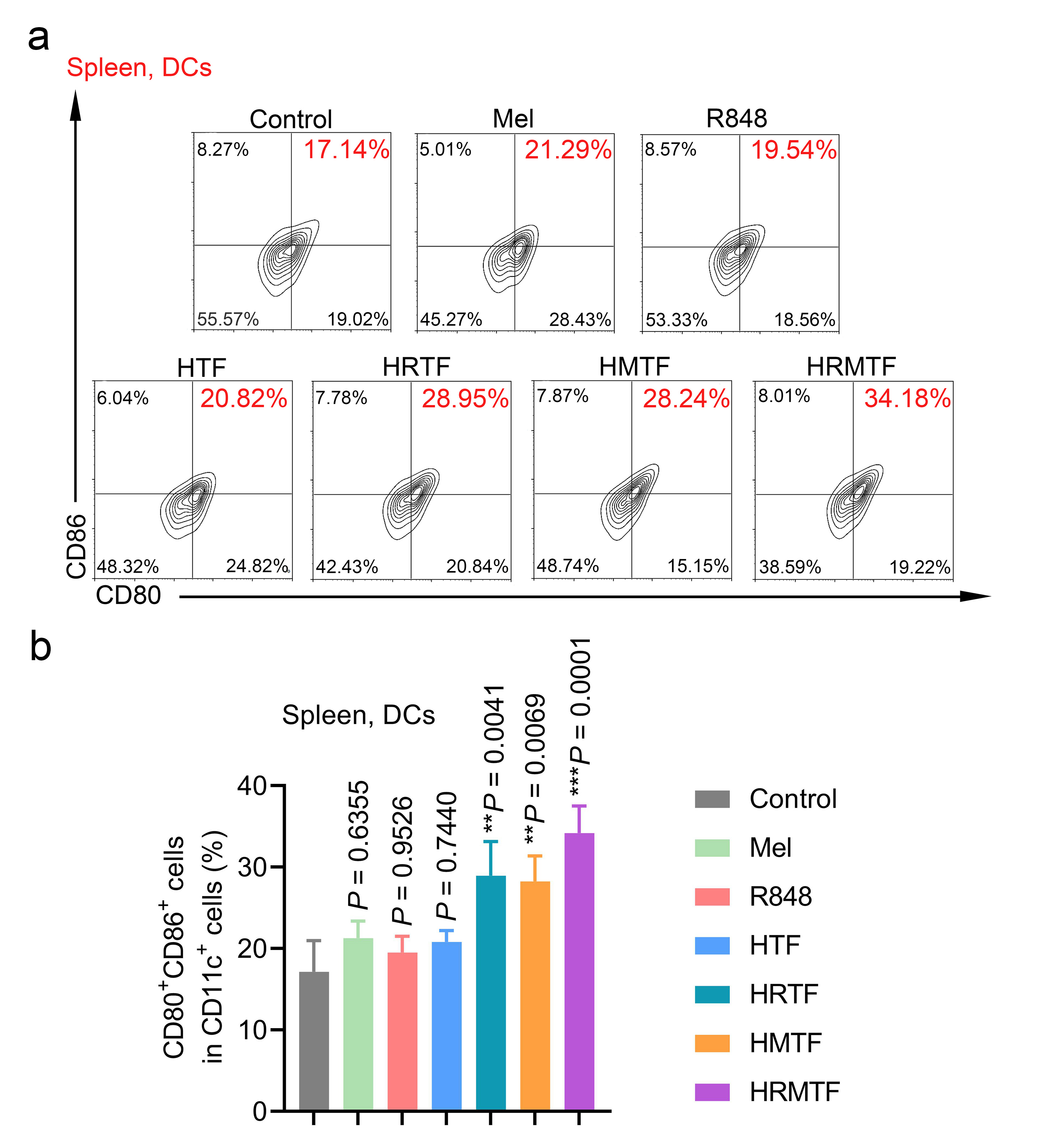


**Figure S20.** Representative flow cytometric plots (a) and corresponding quantification results (b) of mature DCs (CD11c^+^CD80^+^CD86^+^) in the spleens retrieved from the bilateral 4T1 tumor-bearing BALB/c mice 7 d after different treatments. ***P* < 0.01, ****P* < 0.001.


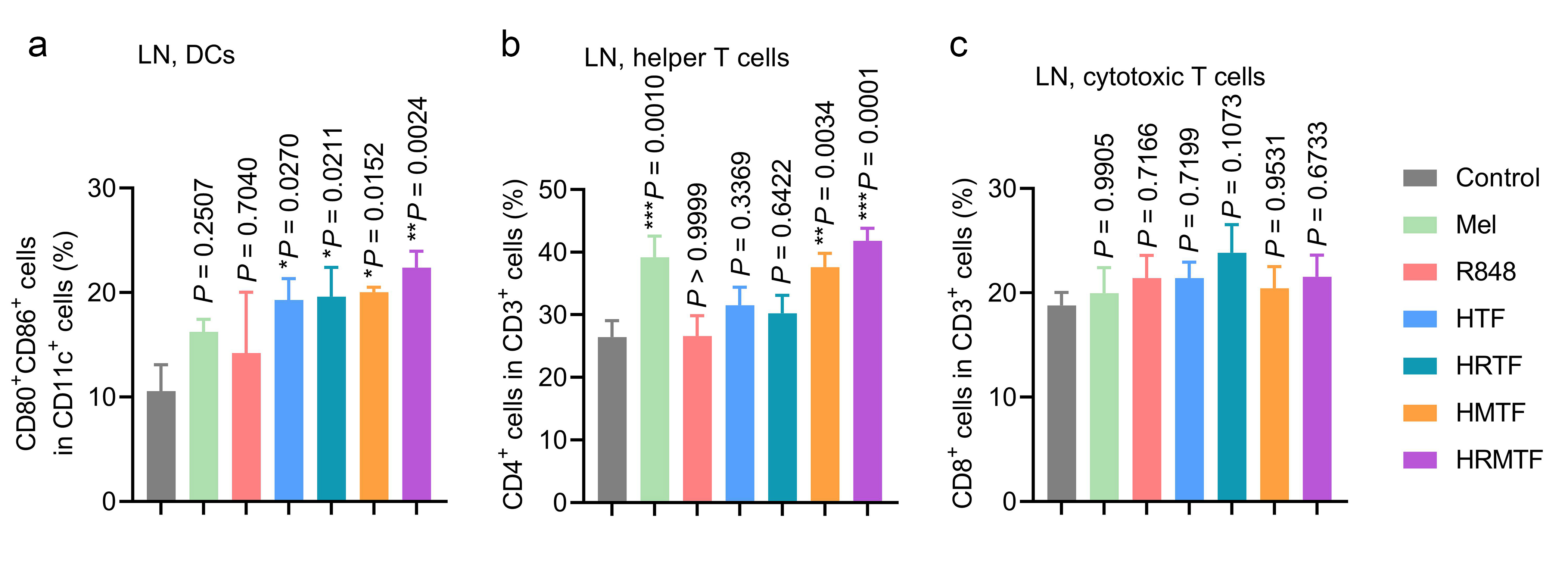


**Figure S21.** Quantification of the flow cytometric results of mature DCs (CD11c^+^CD80^+^CD86^+^) (a), helper T cells (CD3^+^CD4^+^) (b), and cytotoxic T cells (CD3^+^CD8^+^) (c) in the LNs retrieved from the bilateral 4T1 tumor-bearing BALB/c mice 7 d after different treatments. **P* < 0.05, ***P* < 0.01, ****P* < 0.001.


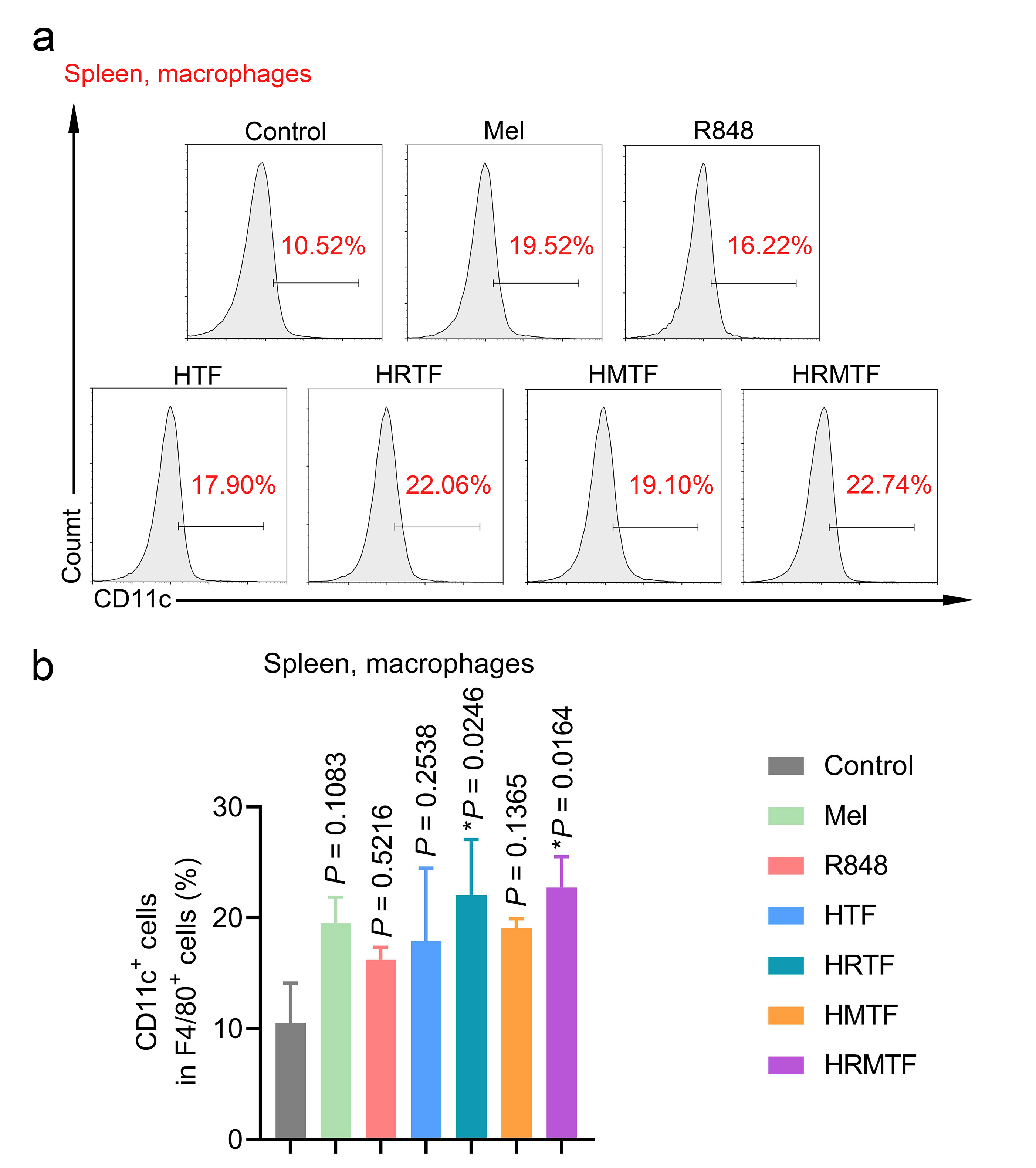


**Figure S22.** Representative flow cytometric plots (a) and corresponding quantification results (b) of M1-like macrophages (F4/80^+^CD11c^+^) in the spleens retrieved from the bilateral 4T1 tumor-bearing BALB/c mice 7 d after different treatments. **P* < 0.05.


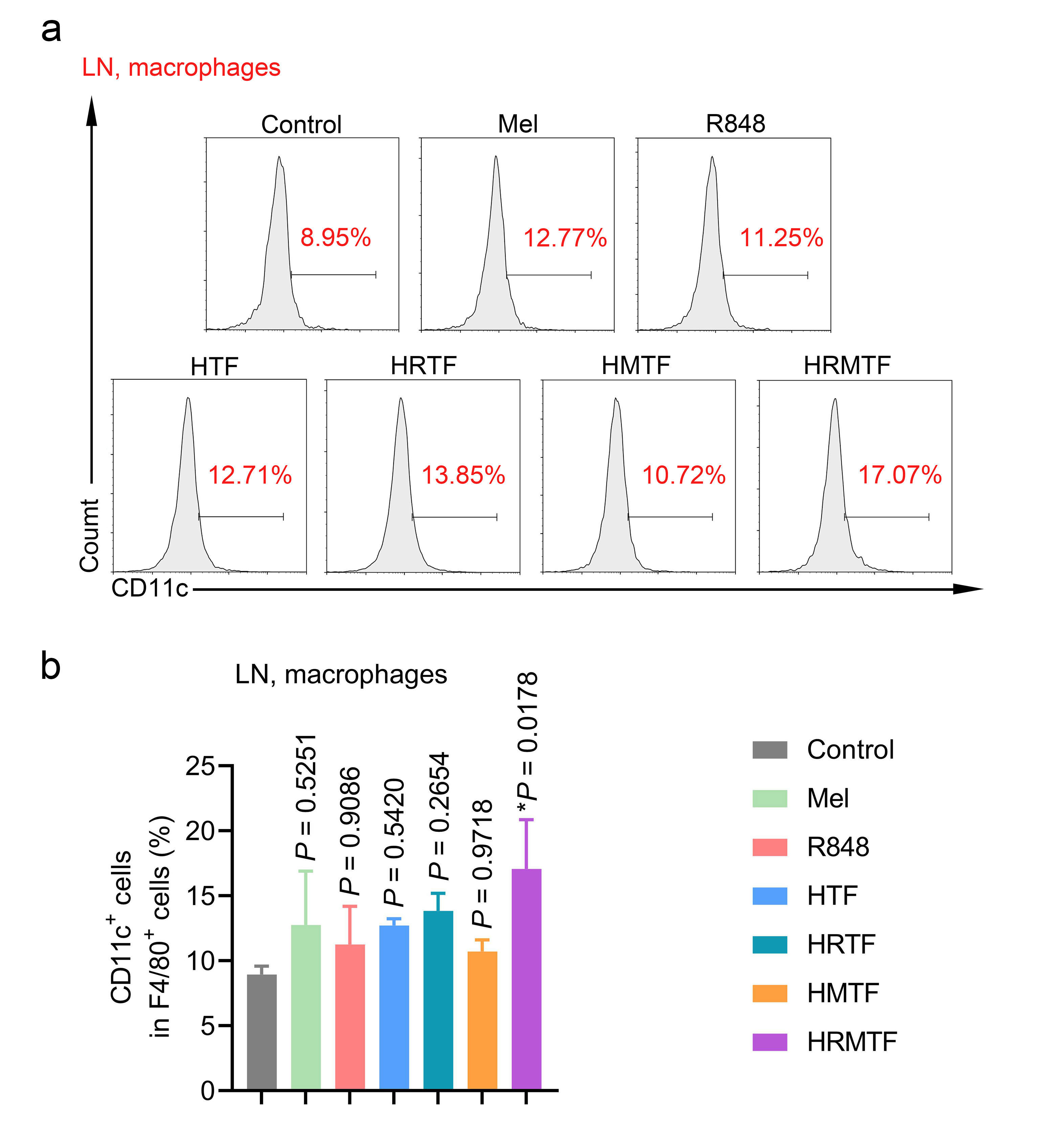


**Figure S23.** Representative flow cytometric plots (a) and corresponding quantification results (b) of M1-like macrophages (F4/80^+^CD11c^+^) in the LNs retrieved from the bilateral 4T1 tumor-bearing BALB/c mice 7 d after different treatments. **P* < 0.05.


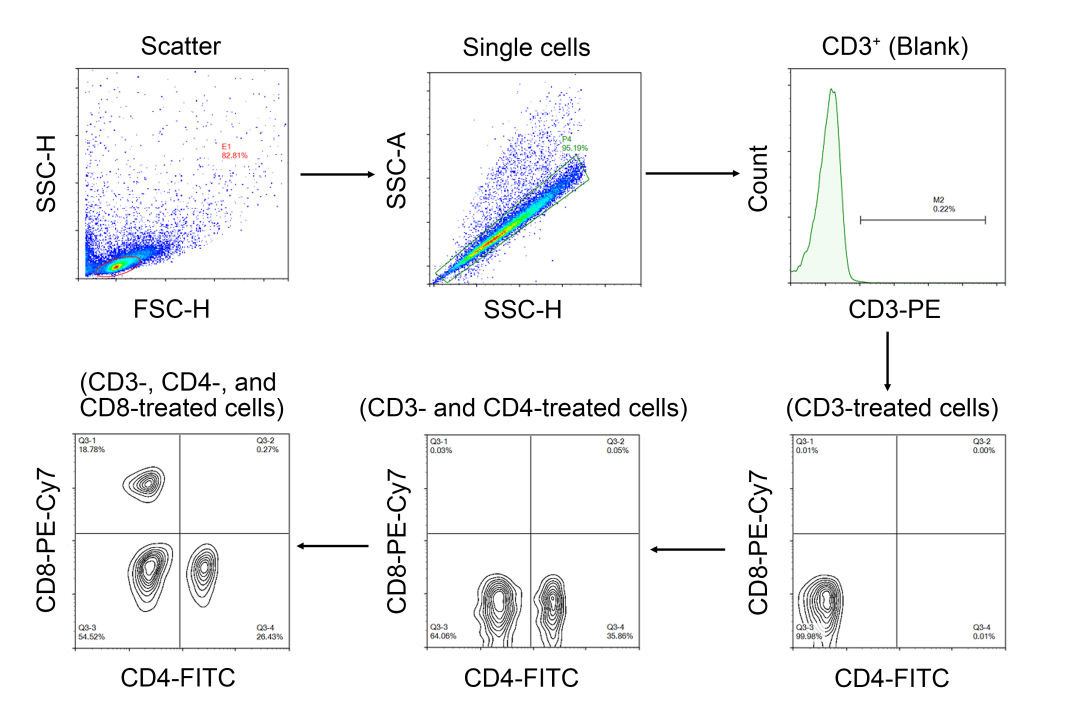


**Figure S24.** Representative flow cytometry gating strategy for CD3^+^CD4^+^ or CD3^+^CD8^+^ T cells. The experiment was performed twice with similar results.


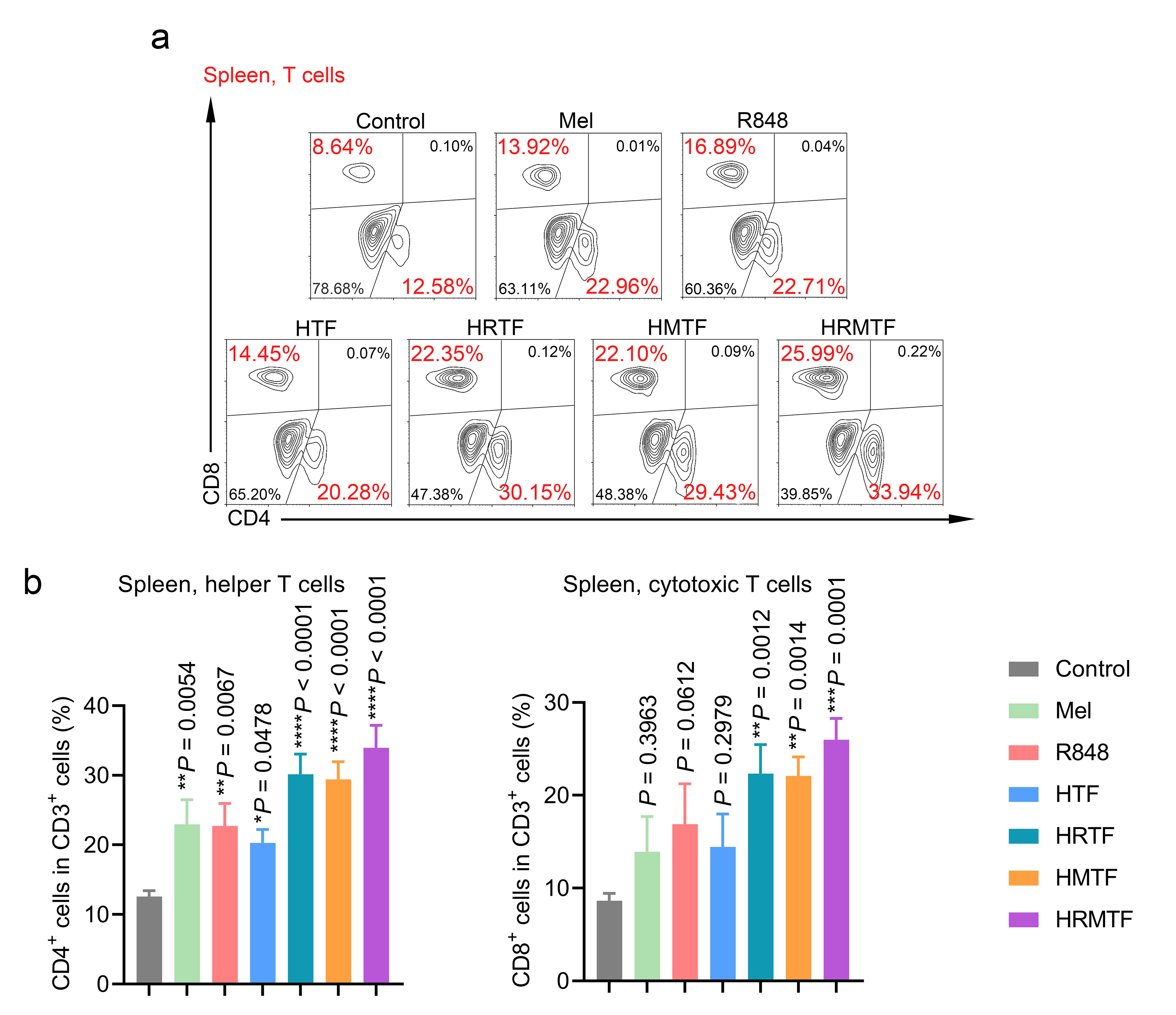


**Figure S25.** Representative flow cytometric plots (a) and corresponding quantification results (b) of helper T cells (CD3^+^CD4^+^) and cytotoxic T cells (CD3^+^CD8^+^) in the spleens retrieved from the bilateral 4T1 tumor-bearing BALB/c mice 7 d after different treatments. **P* < 0.05, ***P* < 0.01, ****P* < 0.001, *****P* < 0.0001.


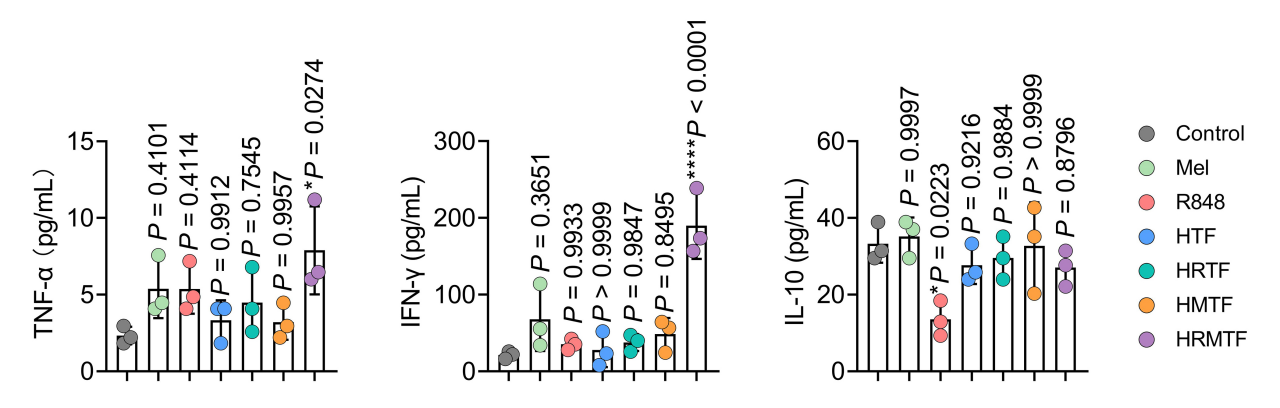


**Figure S26.** Expression levels of TNF-α, IFN-γ, and IL-10 in the serum collected from the bilateral 4T1 tumor-bearing BALB/c mice 7 d after different treatments (analyzed by ELISA). *n* = 3 mice per group. **P* < 0.05, *****P* < 0.0001.
